# A companion to the preclinical common data elements for rodent models of pediatric acquired epilepsy: A report of the TASK3‐WG1B, Pediatric and Genetic Models Working Group of the ILAE/AES Joint Translational Task Force

**DOI:** 10.1002/epi4.12641

**Published:** 2022-10-05

**Authors:** Anna‐Maria Katsarou, Hana Kubova, Stéphane Auvin, Massimo Mantegazza, Melissa Barker‐Haliski, Aristea S. Galanopoulou, Christopher A. Reid, Bridgette D. Semple

**Affiliations:** ^1^ Laboratory of Developmental Epilepsy, Saul R. Korey Department of Neurology Albert Einstein College of Medicine Bronx New York USA; ^2^ Institute of Physiology Academy of Sciences of the Czech Republic Prague Czech Republic; ^3^ Service de Neurologie Pédiatrique, Hôpital Robert‐Debré INSERM UMR 1141, APHP, Université de Paris Paris France; ^4^ Institut Universitaire de France (IUF) Paris France; ^5^ Inserm, LabEx ICST, Institute of Molecular and Cellular Pharmacology (IPMC), CNRS UMR7275 Université Côte d'Azur Valbonne‐Sophia Antipolis France; ^6^ Department of Pharmacy, School of Pharmacy University of Washington Seattle Washington USA; ^7^ Isabelle Rapin Division of Child Neurology, Laboratory of Developmental Epilepsy, Dominick P. Purpura Department of Neuroscience Albert Einstein College of Medicine Bronx New York USA; ^8^ Epilepsy Research Centre, Department of Medicine University of Melbourne, Austin Health Heidelberg Victoria Australia; ^9^ Florey Institute of Neuroscience and Mental Health University of Melbourne Parkville Victoria Australia; ^10^ Department of Neuroscience Monash University Melbourne Victoria Australia; ^11^ Department of Neurology Alfred Health Prahran Victoria Australia; ^12^ Department of Medicine (Royal Melbourne Hospital) The University of Melbourne Parkville Victoria Australia

**Keywords:** brain injury, hypoxic ischemic injury, induction, infantile spasms, seizure, status epilepticus

## Abstract

Epilepsy syndromes during the early years of life may be attributed to an acquired insult, such as hypoxic–ischemic injury, infection, status epilepticus, or brain trauma. These conditions are frequently modeled in experimental rodents to delineate mechanisms of epileptogenesis and investigate novel therapeutic strategies. However, heterogeneity and subsequent lack of reproducibility of such models across laboratories is an ongoing challenge to maintain scientific rigor and knowledge advancement. To address this, as part of the TASK3‐WG1B Working Group of the International League Against Epilepsy/American Epilepsy Society Joint Translational Task Force, we have developed a series of case report forms (CRFs) to describe common data elements for pediatric acquired epilepsy models in rodents. The “Rodent Models of Pediatric Acquired Epilepsy” Core CRF was designed to capture cohort‐general information; while two Specific CRFs encompass physical induction models and chemical induction models, respectively. This companion manuscript describes the key elements of these models and why they are important to be considered and reported consistently. Together, these CRFs provide investigators with the tools to systematically record critical information regarding their chosen model of acquired epilepsy during early life, for improved standardization and transparency across laboratories. These outcomes will support the ultimate goal of such research; that is, to understand the childhood onset‐specific biology of epileptogenesis after acquired insults, and translate this knowledge into therapeutics to improve pediatric patient outcomes and minimize the lifetime burden of epilepsy.

**Key Points**

1. This joint ILAE/AES initiative introduces common data elements (CDEs) related to studies using acquired models of pediatric epilepsy.
2. Case report forms (CRFs) and a companion paper discussing their use are provided for rodent models of early onset epilepsies.
3. Chemical and insult induction models of seizure and epilepsy as applied to pediatric models are described.
4. Future use of these forms may promote standardization of animal experiments to improve reproducibility and facilitate synthesis of findings across laboratories.

## 1 INTRODUCTION

Epilepsy is one of the most common neurologic conditions, affecting approximately 50 million people worldwide^1^ with an international lifetime prevalence of 7.6 per 1000 persons.^2^ Epilepsy across the lifespan exhibits a bimodal distribution, with incidence peaking in childhood and again in the elderly.^3^ Childhood‐onset epilepsies, including both those arising from genetic causes as well as acquired insults, are frequently characterized by drug‐resistant seizures and comorbid cognitive dysfunction, with severe consequences for normal development.^4^ Epilepsies in pediatric populations are a heterogeneous group of disorders that stem from diverse causes, including genetic, structural, metabolic, immune, and infectious conditions, as well as early life insults to the brain^5^; and may be present in the neonatal, infantile, childhood, or adolescent periods of life. However, the cause of disease remains unknown for up to 50% of epilepsy cases.^1^ Regardless of age, epilepsy associated with an acquired brain insult accounts for an estimated one‐third of all epilepsies.^6^

Experimental modeling of acquired epilepsies, including in the pediatric context, is essential to improve our scientific understanding of the fundamental neurobiology that drives epileptogenesis, and to then use this knowledge to develop and test novel therapeutics. However, researchers face many technical challenges in this context, whereby seemingly subtle differences (both known and unknown) in experimental procedures used by different groups may render quite different experimental outcomes. Such heterogeneity makes it very difficult to compare findings between laboratories, conduct meta‐analyses, or draw solid conclusions. To address this, we have developed a set of common data elements (CDEs) for pediatric acquired epilepsy models in rodents. CDEs provide a framework on which to improve the standardization of experimental designs across laboratories, allowing for different laboratories to replicate experiments, validate, and ultimately improve the ability to translate insights gained from rodent models into the clinic.^7^, ^8^

A wide range of pathogenic mechanisms contribute to the etiology of acquired epilepsies in pediatric populations.^5^ Many of the pathologies associated with acquired epilepsies may have a known or suspected genetic component, as well.^9^ Acquired epilepsies may be developmental in origin—such as vascular malformations, cortical dysplasia, or hippocampal sclerosis due to congenital lesions—resulting in symptomatic structural‐related epileptic syndromes. Pediatric epilepsies that are associated with such structural malformations are often characterized by genetic factors; for example, mutations in mammalian target of rapamycin and its downstream signaling pathways are a known cause of focal cortical dysplasia.^10^, ^11^ Genetic models of genetic etiology epilepsies will be discussed and addressed in a separate companion paper associated with genetic‐specific Case Report Forms (CRFs). Perinatal and postnatal stroke, hypoxic–ischemic injury, intracranial hemorrhage, traumatic brain injury (TBI), and maternal or neonatal infection to the central nervous system (CNS) (e.g., herpes simplex encephalitis and Zika virus) are also common causes of acquired epilepsy.^12^, ^13^, ^14^ Determination of the relevant etiology for a pediatric patient's epilepsy has important implications for seizure management and treatment options, such as surgery.

When considering the spectrum of acquired epilepsy models, particularly those presenting during a pediatric or juvenile age period, we have focused on two broad categories: (1) physical induction models and (2) chemical (or pharmacological) induction models. CDE tables with key parameters central to the generation and characterization of these models were developed, accompanied by specific CRFs containing the descriptors for the CDE. Overarching and complementary to these specific CRFs is a core CRF for pediatric models of acquired epilepsy. Together, our aim was to provide investigators with useful/usable templates to facilitate the harmonization of the collection of essential data in rodent models across laboratories, with the goal of improving scientific rigor and reproducibility of studies in the field. This companion manuscript will describe the development of these CRFs and CDEs for pediatric acquired epilepsy models and will provide background information describing the importance of the required elements.

## 2 PROCESS OF GENERATING CRFs AND CDEs

The Working Group 1B “Pediatric and Genetic Models” of the ILAE/AES Joint Translational Task Force was established in 2019. The working group was comprised of epilepsy researchers from across Europe, the United States, and Australia, who were invited by the ILAE/AES Leadership in efforts to ensure a balanced representation of geographical origin, gender, and career stage, as well as a breadth of expertise related to the topic. The group met regularly in a virtual manner to initially develop the CRFs for various genetic and acquired models of epilepsy. The CRFs were then converted into CDEs, analogous to previous preclinical CDEs generated as part of the ILAE/AES Joint Translational Task Force TASK3 working groups.^7^, ^15^, ^16^, ^17^, ^18^

Each element was discussed, and a consensus on its importance and inclusion was reached during face‐to‐face virtual meetings of the Working Group. Draft CRFs were generated off‐line by B.D.S. and A.K., then circulated within the Working Group for review and multiple rounds of revisions, including discussion of key elements via virtual meetings. Priority levels assigned to the CRF components were proposed by B.D.S. and A.K., then discussed and agreed upon by consensus within the Working Group. Published literature on rodent epilepsy model research was consulted and incorporated where relevant. Final draft documents were then reviewed by other TASK3 Working Groups for feedback and refinement, prior to finalization and approval by the ILAE and AES Leaderships.

## 3 OVERVIEW AND UTILIZATION OF PEDIATRIC ACQUIRED EPILEPSY CRFs

**FIGURE 1:**
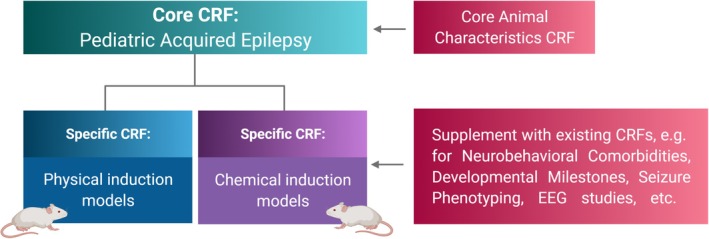
Case report forms (CRFs) for pediatric acquired epilepsy. A Core CRF provides a centralized source of key information that may be common across several animals, including the animal species, strain and origin, and study design elements including model choice and outcome measures. This *Core CRF* should be accompanied by the Core Animal Characteristics CRF, which covers pertinent information regarding housing conditions. One of two *Specific CRFs* can then be selected and completed for a specific experimental procedure, to document specific information regarding the physical or chemical induction model parameters. Both core and specific CRFs are linked by each animal's unique identification number, and may be supplemented by existing CRFs where appropriate, for example, to document model‐specific neurobehavioral observations^17^ and seizure or sleep–wake cycle phenotypes.^18^ Made in Biorender.com

In the present companion manuscript and associated CDEs and CRFs, we focus on rodent models of pediatric acquired epilepsies. A single *Core CRF* was designed as a common document for an experimental study or cohort of animals (Table 1). In conjunction, two *Specific CRFs* have been designed to describe a specific experimental procedure to induce an acquired epilepsy model in a sub‐cohort of experimental animals (Table 2, Table 3). To minimize repetition with existing CRFs or prior reports addressing methodological issues in seizure or epilepsy phenotyping experiments (e.g., regarding collection and interpretation of behavioral observations, EEG data, physiological data and pharmacology), we have referred to these where applicable, and recommend the researcher to refer to these resources where appropriate^7^, ^15^, ^16^, ^17^, ^18^, ^19^, ^20^ (Figure 1). CRFs will be available in an electronic format to allow for linkage between documents. Of note, each component of the CRF is designated a priority level—High (H), Intermediate (I), or Low (L)—to guide the researcher about the relative importance of recording each data point. For example, identification information about the litter (name/number) is considered high priority data to collect; whereas identifying details about the parents may be considered intermediate priority or optional to include. We propose that all available data are captured wherever possible; however, this prioritization system will allow for capture of the most essential data elements if less detailed CRFs are used. The purpose of this project was to generate a comprehensive resource which individual investigators in different scenarios can tailor to their own use.

The CDEs presented here apply to immature rodents—rats and mice—in the first few days or weeks of life, as the most common experimental models for pediatric epilepsy research. The immature age group may also be referred to as pediatric or juvenile in some studies. Age‐ and sex‐specific considerations have been incorporated where appropriate, keeping in mind age‐ and sex‐related developmental milestones that parallel or differ between species (rodents and humans) as they may be important to interpret clinical relevance, as compared and reviewed in depth elsewhere.^21^, ^22^, ^23^ For example, postnatal day 7–11 in rats and mice is typically considered to be equivalent to a full term newborn baby,^21^ and is the most common age range used to model brain insults in the perinatal and early postnatal period that promote epileptogenesis, such as hypoxia.^24^ However, there are significant species differences in the trajectory of maturation of specific developmental processes that need to be considered in developmental studies.^24^ CDEs specific to immature animals and distinct from those of adult animals are necessary, as responses to epileptogenic insults and their consequences may be dependent on the developmental stage of the animal. For example, developmental age and sex can influence seizure susceptibility/threshold for seizure onset, the impact of seizures on the brain, as well as efficacy versus toxicity of antiseizure drugs.^22^, ^23^, ^25^, ^26^ We also focus on models that are frequently applied to immature rodents to recapitulate common conditions seen in young children, such as febrile seizures.

### 3.1 Core CRF—Rodent models of pediatric acquired epilepsy

CRF File name: 1. Core CRF module Rodent models of pediatric acquired epilepsy.docx

CDE File name: 1. Core CDE_chart Rodent models of Pediatric Acquired Epilepsy.xlsx (Supporting information)

The *Core CRF* has been designed to be applicable to a single cohort of experimental animals (Table 1). The Core CRF encapsulates metadata of experimental parameters that are critical to the use of acquired epilepsy models in immature rodents. Specific CRFs are to be used in conjunction with this core CRF to describe the experimental procedures (i.e., induction model details) applied to individual cohorts or sub‐cohorts of animals (Figure 1). The date captured here refers to the date at which the form was completed. In contrast, the date of model induction is recorded elsewhere in the CRF.

**TABLE 1:** Case report form: CORE CRF—Rodent models of pediatric acquired epilepsy

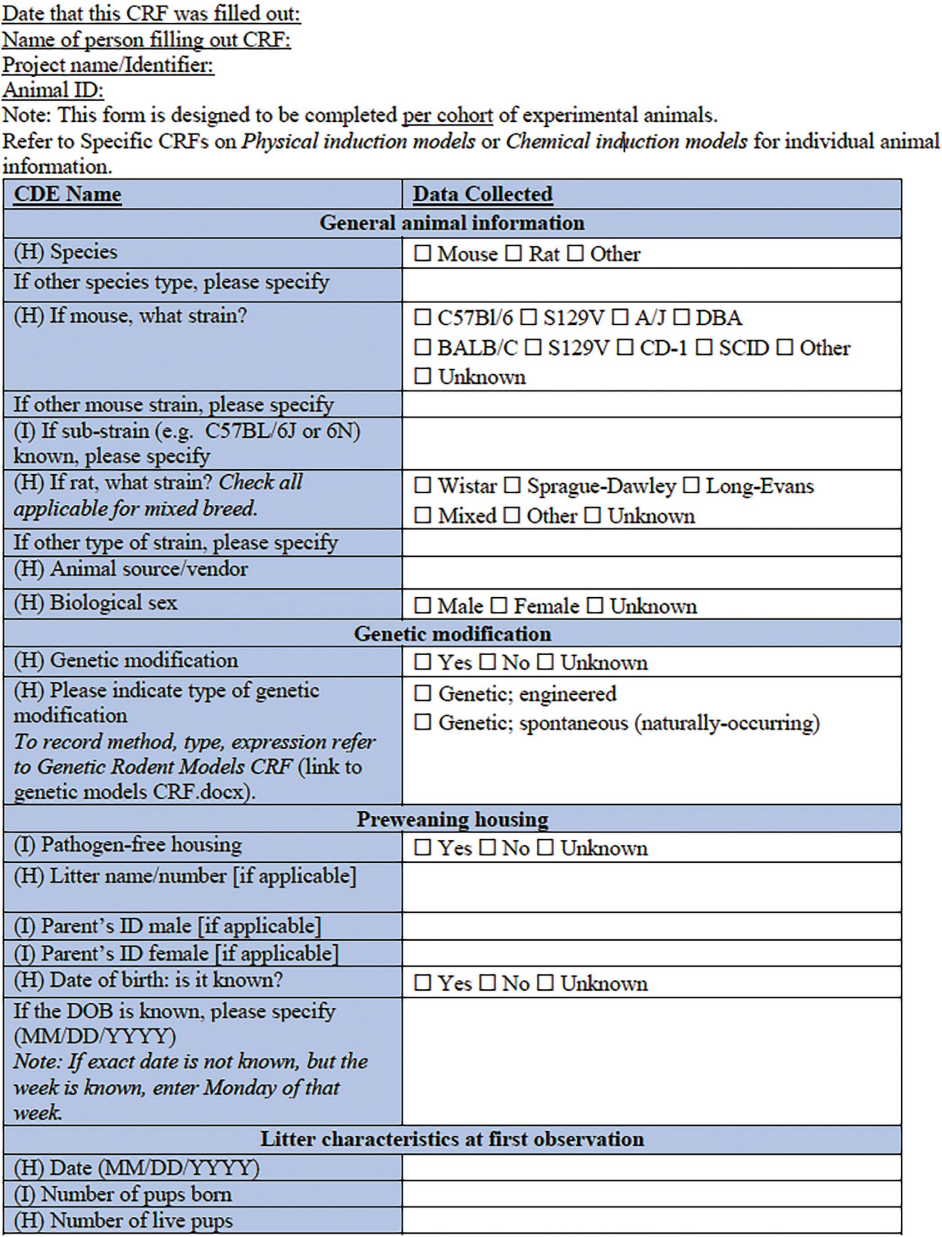
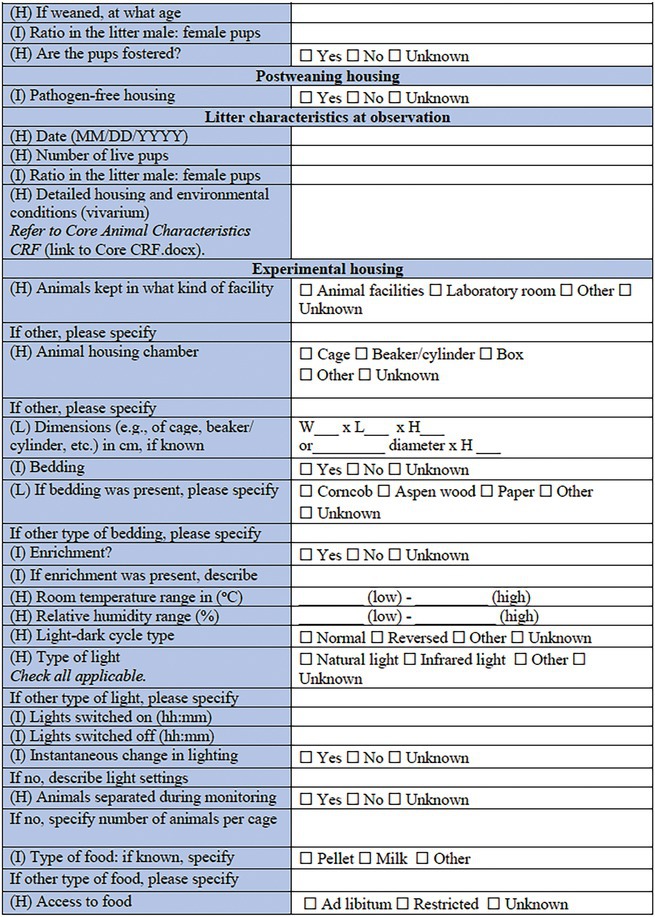
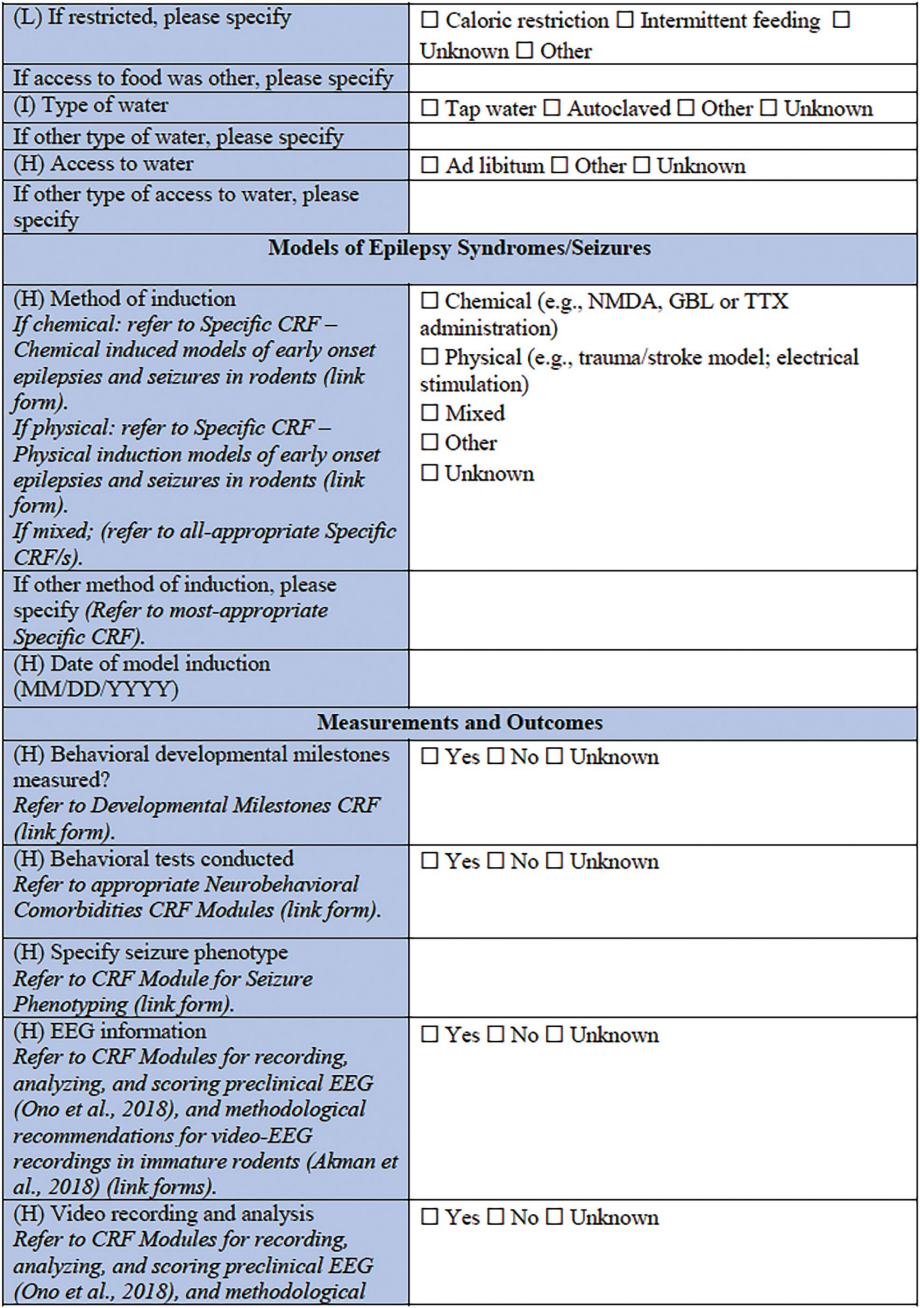
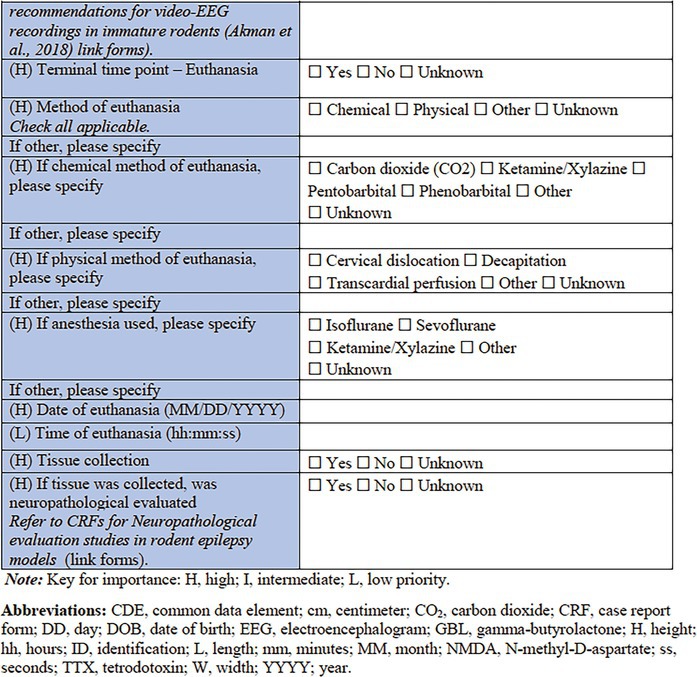

#### 3.1.1 Core CRF: General animal information

This section is designed to capture key information regarding the identity of the animal cohort, including the species, sex, strain, and animal source/vendor. Additional information pertaining to individual animals regarding the animal source and related information can be captured by linking to the *Core Animal Characteristics CRF*,^7^ which we recommend utilizing in association with the model‐specific CRFs described below.

#### 3.1.2 Core CRF: Genetic modification

There is increasing evidence that genetic factors may contribute to vulnerability to epilepsy after an acquired insult.^9^, ^27^, ^28^ As such, we anticipate seeing an increase in the number of experimental models that likewise incorporate both genetic risk‐related genotypes along with acquired epilepsy induction models. If the animals used are genetically modified (e.g., transgene, knockout), refer to the *Genetic Rodent Models CRF* to capture pertinent information regarding the specific genetic change, the human disease/syndrome associated with the genetic change, and the procedure for model generation/maintenance.

#### 3.1.3 Core CRF: Pre‐ and postweaning housing

Of particular importance for pediatric models of epilepsy, this section requests data regarding litter characteristics (e.g., number of pups born; number live until X day; ratio of male: female; and any fostering, if known). This information should be noted both preweaning (at first observation of the litter) as well as postweaning, to record investigator‐defined group housing conditions. A more detailed description of the housing conditions is often essential information to ensure consistency across experiments, which can be captured in a linked document *Core Animal Characteristics CRF* (e.g., cage sizing, covers, rack/holding system, bedding material, enrichment if present, room temperature/humidity, lighting, and diet).^7^ The date of birth should be considered as postnatal day 0 (“P0”) and therefore the day following birth will be P1.

#### 3.1.4 Core CRF: Experimental housing

While many details regarding the vivarium housing conditions can be readily captured in the published above‐mentioned *Core Animal Characteristics CRF*, it is pertinent to separately consider the environmental conditions during model induction. This section is designed to clarify where precisely the animals are maintained during experimentation, and the housing circumstances during the experimental period, including access to food and water, room lighting and temperature, and group versus individual animal housing.

#### 3.1.5 Core CRF: Models of epilepsy syndromes/seizures

Several methods of induction are listed. For solely genetic‐based models of epilepsy, please refer to *Genetic Rodent Models CRF* (and indeed, use it instead of this form). For chemical‐induced or physical‐induced models of early life epilepsies and seizures in rodents, refer to the appropriate linked Specific CRF (see below). If the model is mixed (e.g., a genetic mouse model that confers seizure susceptibility, combined with a secondary physical‐induction model such as hypoxic–ischemic injury), refer to the most appropriate Specific CRFs in combination. Physical induction models include models of TBI, ischemic stroke, hypoxic–ischemic or hypoxia‐alone, hyperthermia, and electrical/optogenetic or other modes of stimulation‐induced status epilepticus (SE). Chemical induction models include models of infantile spasms (IS; e.g., by doxorubicin/lipopolysaccharide/p‐chlorophenylalanine administration); chemically induced SE (e.g., by kainic acid administration); and infection‐like models (e.g., maternal immune activation).^29^, ^30^ Embryonic insults such as in utero irradiation, embryonic freeze lesions, or in utero exposure to methyl‐azoxy‐methanol acetate (MAM) are not included here, as we chose to focus on perinatal and postnatal insults. We also acknowledge that classification of models into purely physical versus chemical induction is a somewhat simplistic approach. For example, hypoxic injury models could be classified as either physical or chemical; however, we have chosen to list this model here as a physical induction to readily allow for its combination with an ischemic challenge (another physical induction model), as it is commonly performed.

#### 3.1.6 Core CRF: Measurements and outcomes

For an experimental cohort of animals, identify the relevant outcome measurements incorporated into the study design. For immature rodent models, we recommend consideration of developmental milestones (refer to *Developmental Milestones CRF*),^17^ to include a battery of assessments for growth, motor function, and coordination of young rodents to assess the effects of seizures, epilepsy, and their comorbidities on normal development. Additional behavioral testing, particularly at the experimental end point, may incorporate assessments for general activity, sensorimotor, neurocognitive and psychosocial outcomes, and these should also be incorporated and linked (refer to appropriate *Neurobehavioral Comorbidities CRF* modules).^17^ Detailed observations of seizure and seizure‐like behaviors should be captured using the *Seizure Phenotyping CRF*. Video‐EEG (vEEG) information is also integral to model characterization. The latter can be described using the CRF modules for rodent vEEG studies.^18^ More information on methodological and interpretation issues pertaining to vEEG studies in immature rodents can be found in the TASK1‐WG2 report.^19^

Finally, some records regarding the experimental endpoints are sought, including the method of euthanasia (if performed); whether tissue was collected for postmortem analysis, and whether neuropathological evaluations were performed. Further details regarding tissue collection and subsequent analysis can be captured using a relevant complementary CRF.

### 3.2 Specific CRFs

Two *specific CRFs* have been developed for pediatric acquired epilepsy models in rodents: (1) Physical induction models, and (2) Chemical induction models. These are designed to be completed for specific experimental procedures, and are linked with the cohort‐based Core CRF via the cohort identification number listed above the table.

### 3.3 Specific CRF 1: Physical induction models (Table 2)

CRF File name: 2. CRF module Physical induction models.docx

CDE File name: 2. CDE_chart Physical induction models.xlsx (Supporting information)

**TABLE 2:** Case report form: Specific CRF 1—Physical induction models of early onset epilepsies and seizures in rodents

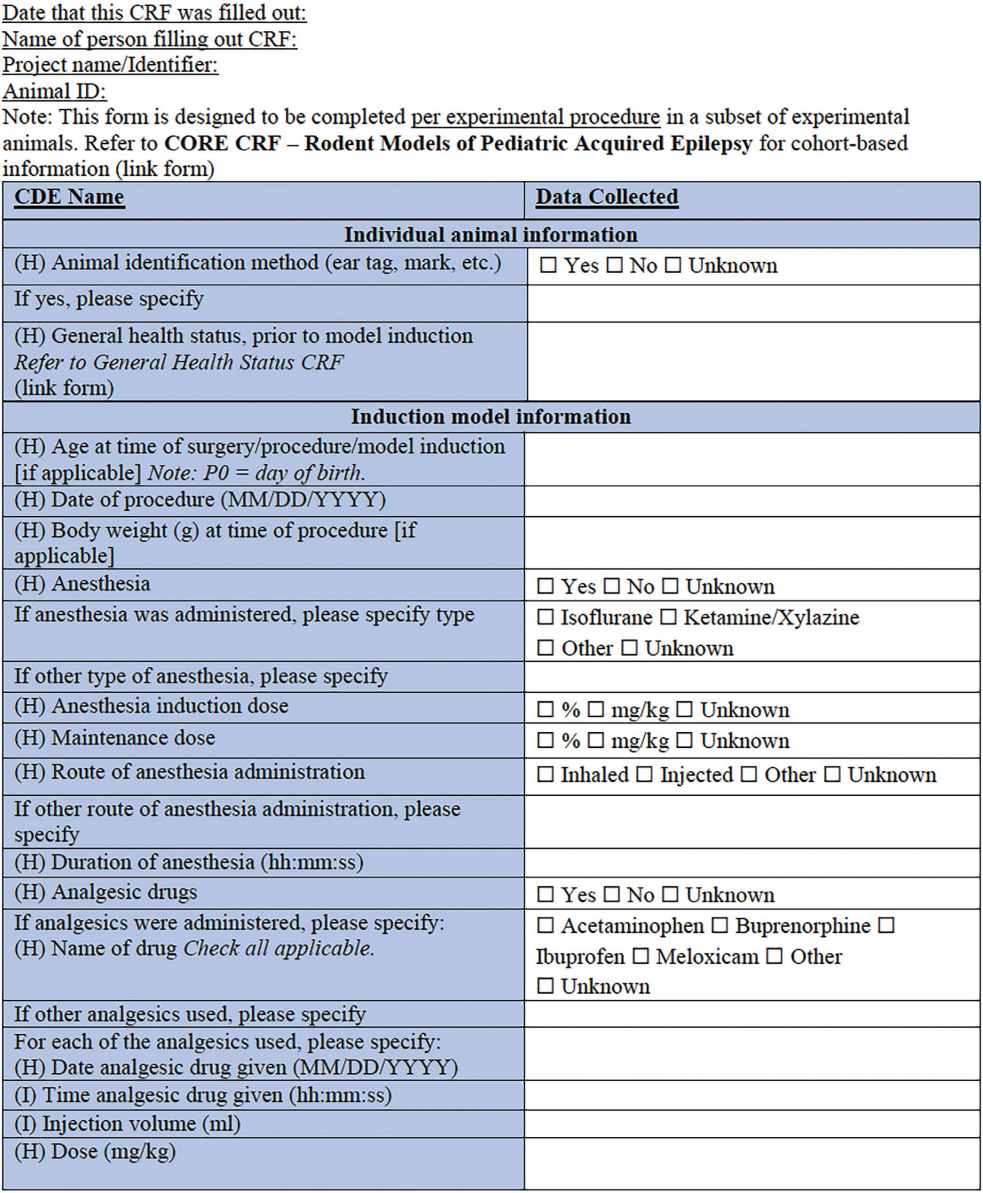
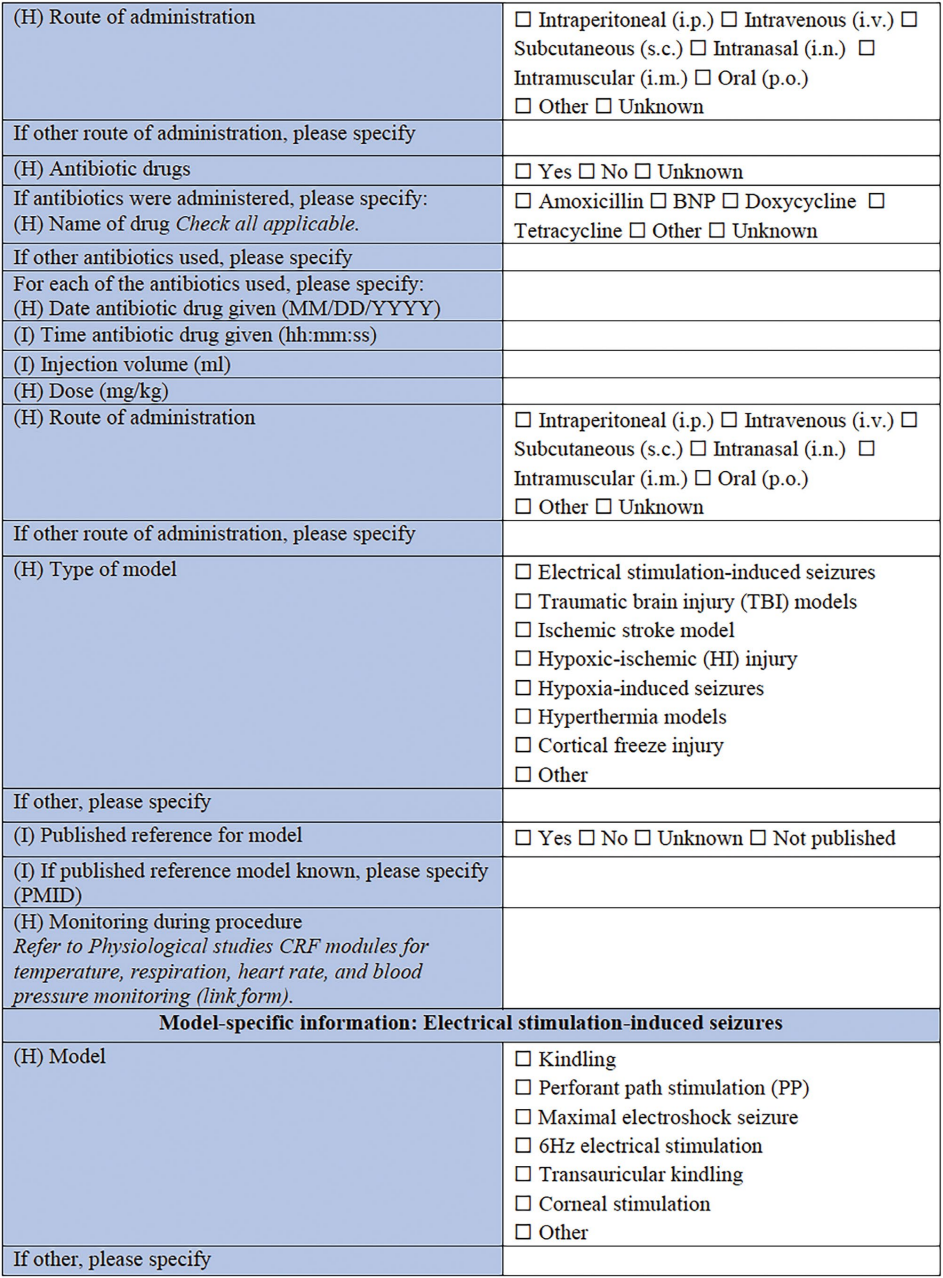
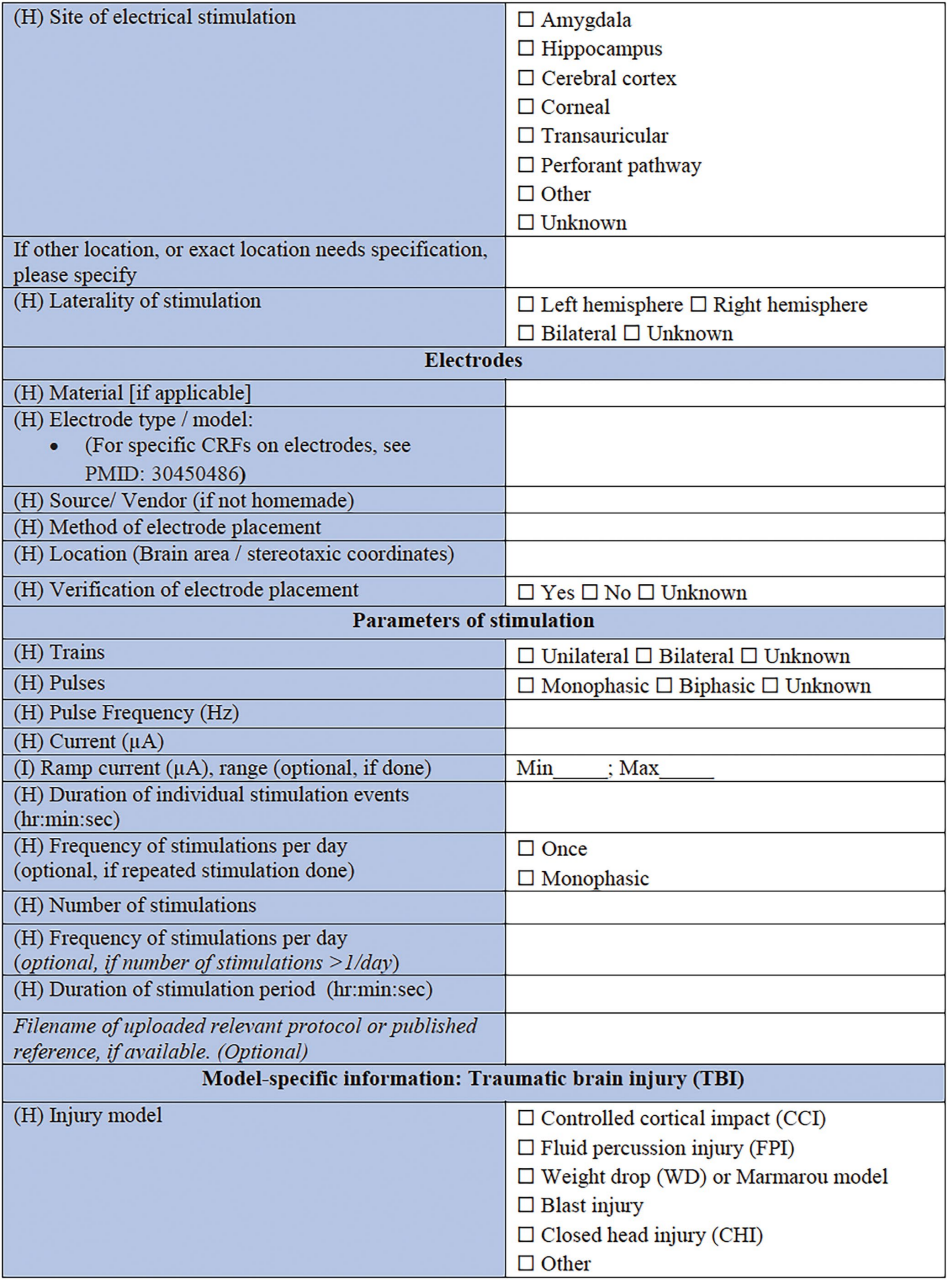
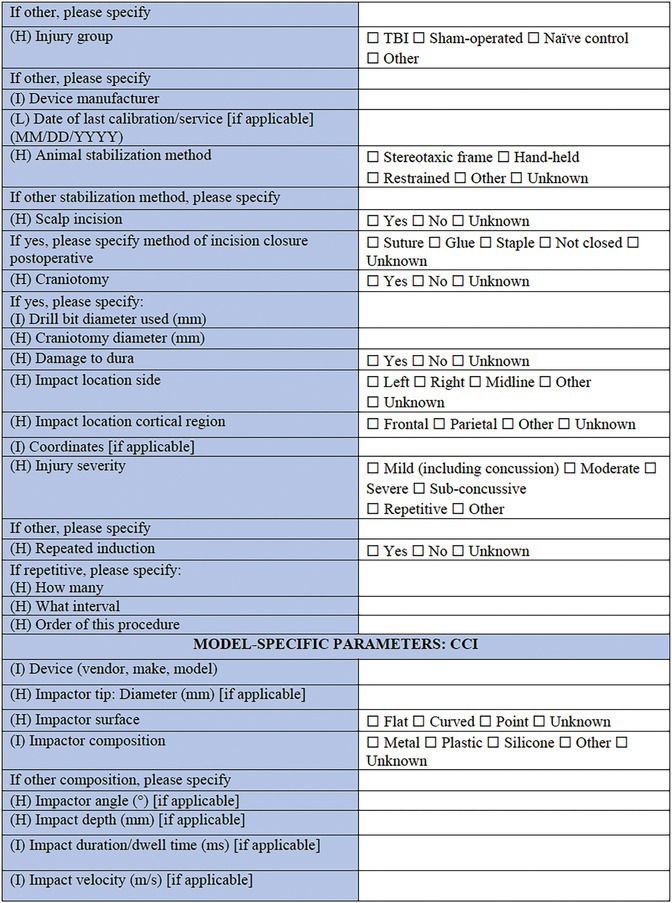
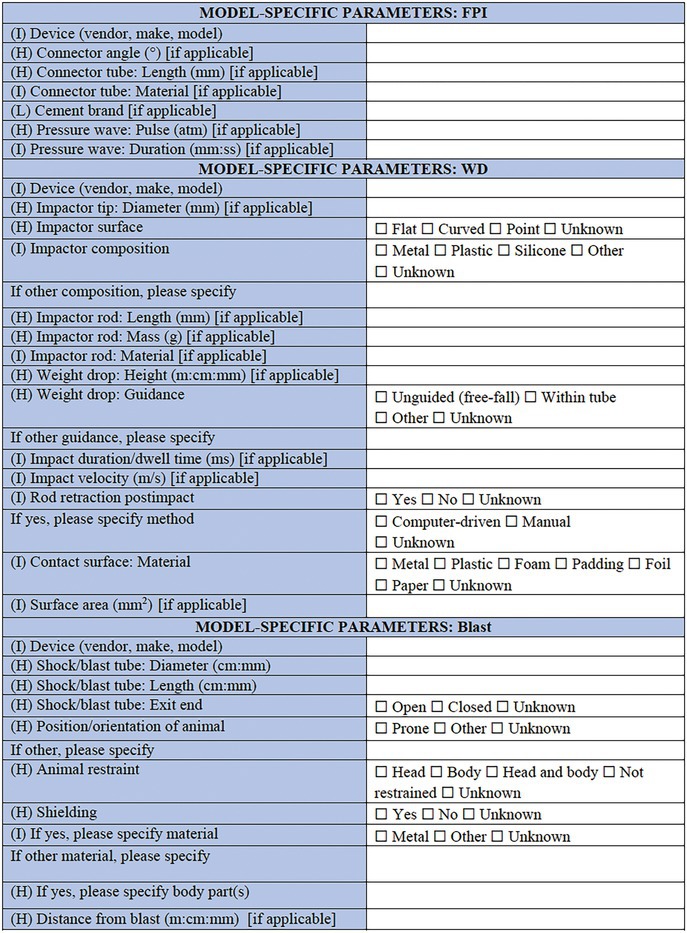
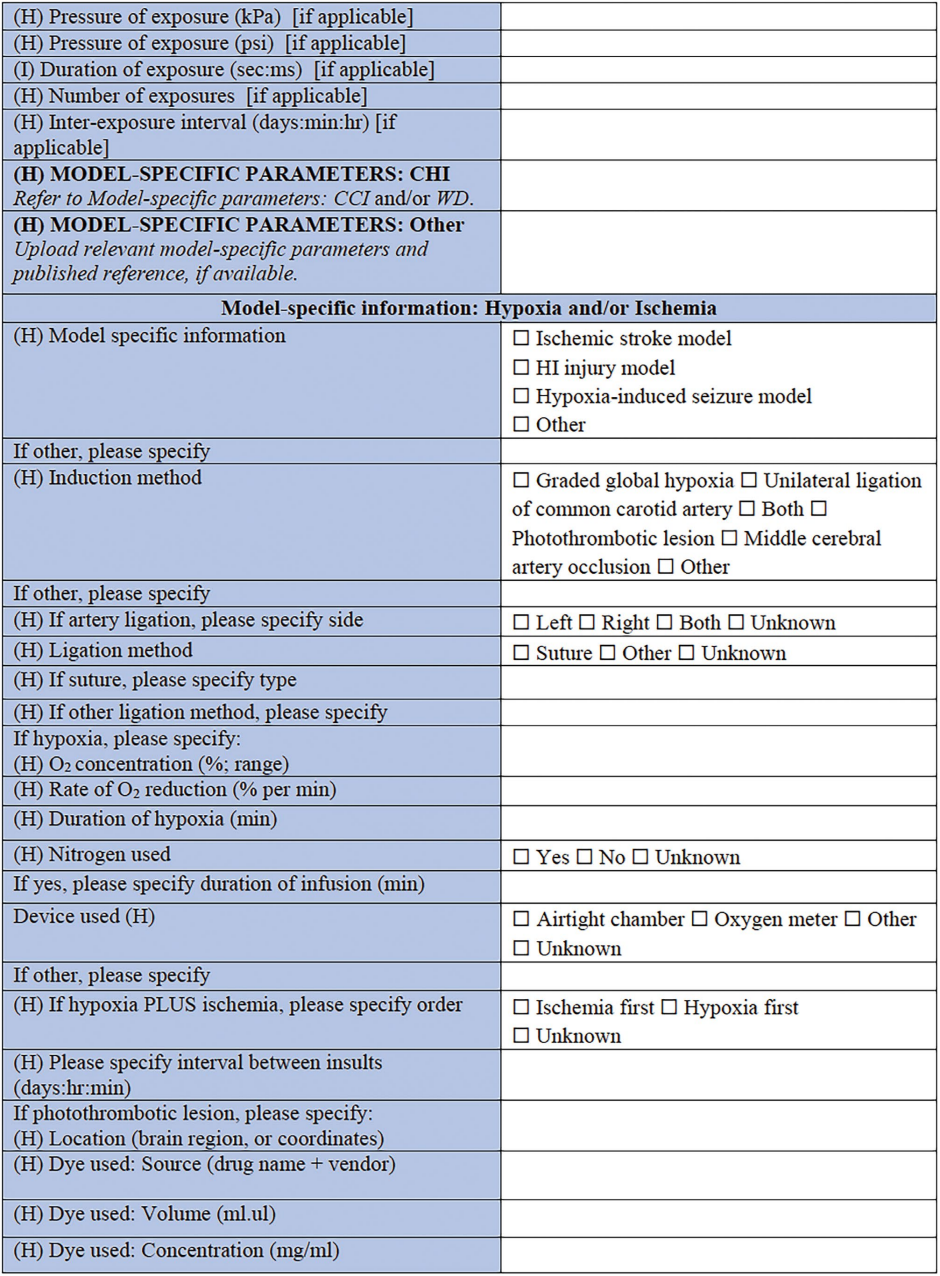
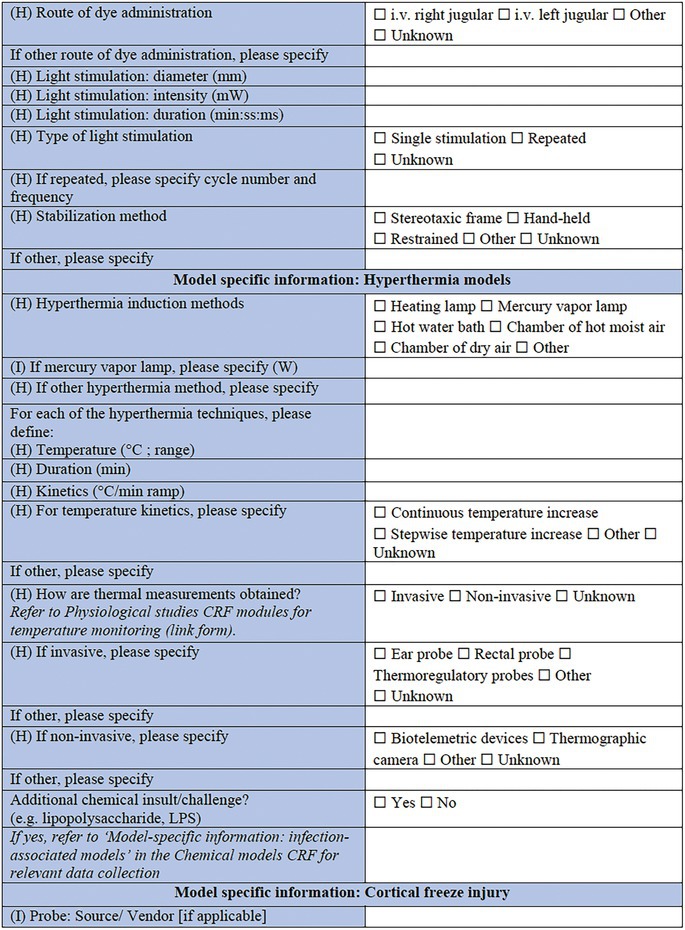
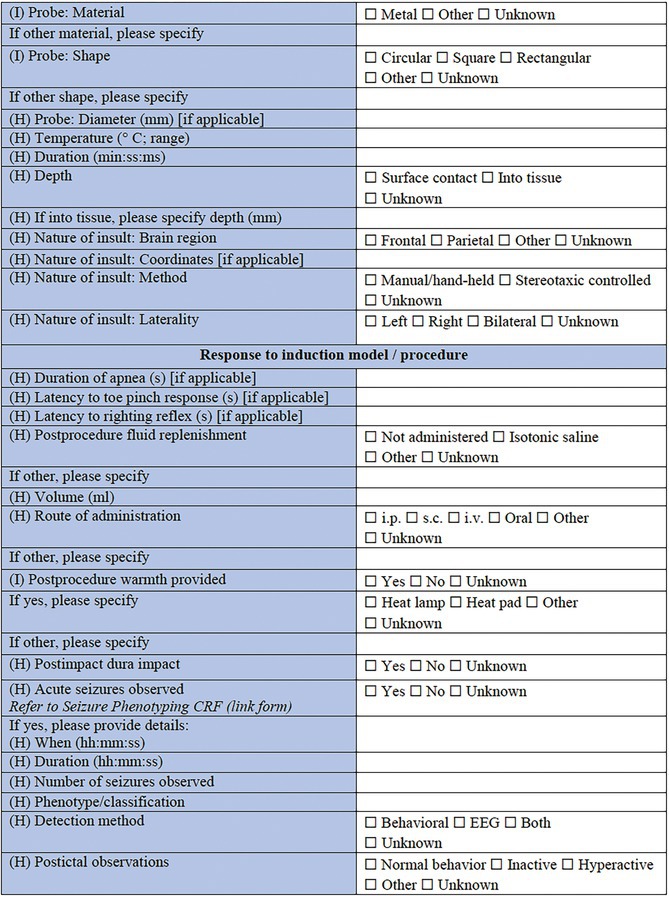
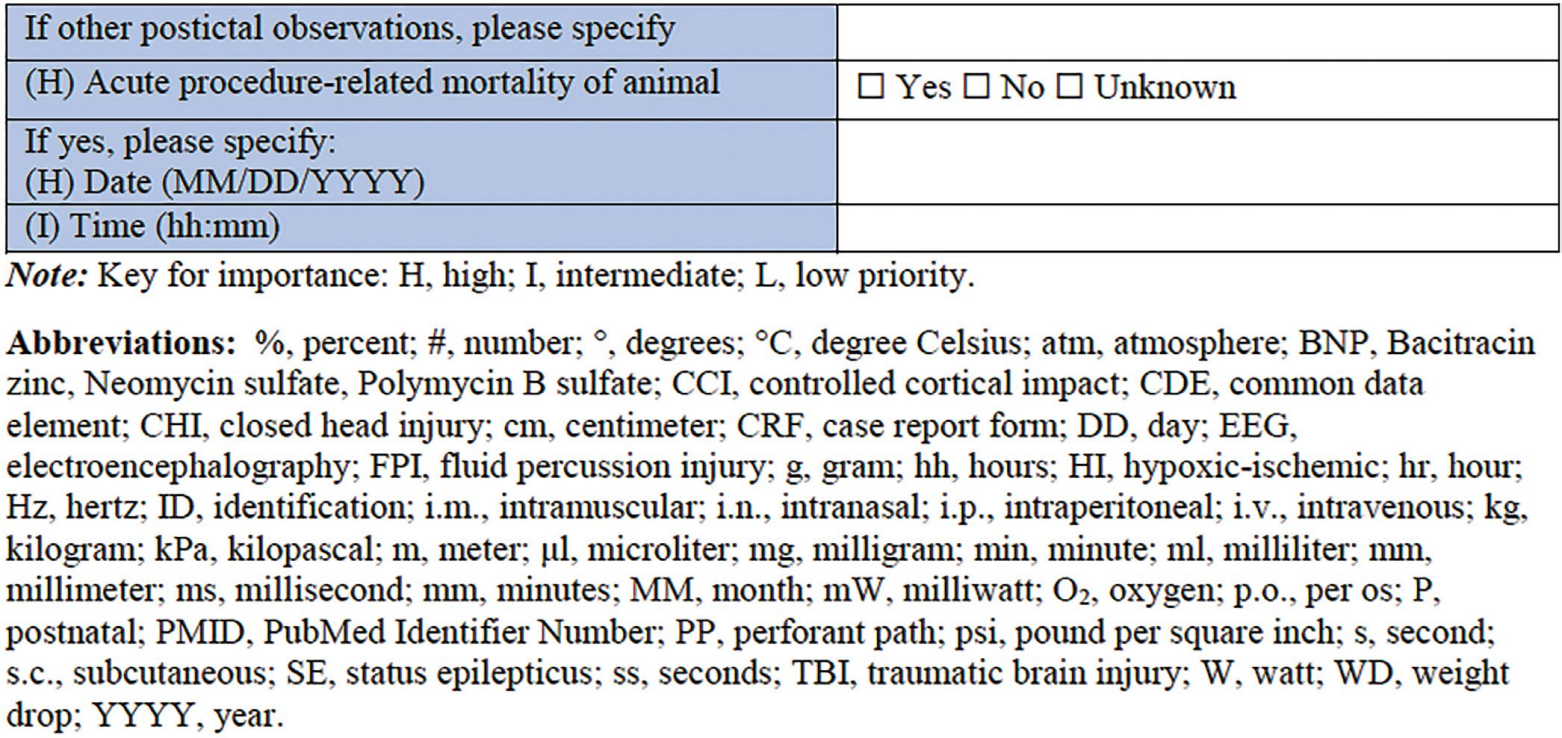

#### 3.3.1 Rationale

This specific CRF is designed to cover rodent models of pediatric acquired epilepsy generated by physical induction methods during the perinatal period or early postnatal life. This category encompasses models that involve surgical intervention to induce a TBI, ischemic stroke, hypoxic–ischemic injury, or cryolesion, a hyperthermic environment, or electrode‐mediated electrical stimulation to induce SE.

#### 3.3.2 Individual animal information

Overlapping with the Core CRF, this section captures key information regarding the identity of the cohort and individual, including the species, strain, and date of birth. Additional information pertaining to an individual animal's health and condition prior to model induction can be captured in the linked *General Health Status CRF*.^7^

#### 3.3.3 Induction model information

This section records information regarding the specific features of the induction model. It is critical to identify the age at the time of model induction, as well as the body weight of the animal at the time of the procedure, surgery, or model induction, and the date that this takes place. For models that require surgery and anesthesia, specify the type, dose, duration, and administration route of anesthesia, for induction as well as maintenance stages. If pain medications and antibiotics are used, they should be listed in the relevant sections along with details of the date and time they are administered, the injection volume (ml/kg body weight), the dose (mg/kg), and the route of administration. Monitoring performed during and after the surgical procedure can additionally be documented using the *Physiological Studies CRF* modules (e.g., temperature, respiration, heart rate, and blood pressure monitoring details).^15^

The type of physical model should then be noted, according to the following categories: electrical stimulation‐induced seizures; TBI models; ischemic stroke models; hypoxic–ischemic (HI) injury or hypoxia‐induced seizures; hyperthermia models; neonatal freeze injury, or other. Where possible, a published reference for the chosen model should be listed using the PubMed Unique Identifier number (PMID). Subsequent sections of the form capture key information of importance to each of the models, noting that only the relevant section for the chosen model needs to be completed for an animal and cohort.

#### 3.3.4 Model‐specific information: Electrical stimulation‐induced seizures

Electrical stimulation comprises some of the most common experimental models used to study seizure susceptibility (i.e., maximal electroshock seizures, 6 Hz electrical stimulation seizures), reproduce the development of progressively more severe seizures (i.e., kindling), or elicit SE and subsequent chronic spontaneous seizures.^31^, ^32^, ^33^, ^34^, ^35^ For example, in the classical kindling stimulation model, animals undergo repeated (i.e., daily or twice‐daily or more frequently in infantile pups) electrical stimulations over the course of days via implanted electrodes into seizure‐prone brain regions (e.g., hippocampus or amygdala) to model human limbic seizures.^36^ Electrical stimulation might also be applied to cornea or via ear clip topical electrodes.^37^, ^38^, ^39^ Local electrical brain stimulation, like the stimulation of the perforant pathway, can also result in the induction of SE and may induce spontaneous seizures.^40^ The location of the stimulus (laterality, brain region, and/or stereotaxic coordinates), and nature of the stimulating electrodes (metal composition, resistance [in Ohms, if known] and source) are critical details to record. Then, the parameters of the stimulation (unilateral or bilateral stimulation; monophasic or biphasic pulses; frequency, pulse, and train duration; ramp current range and current amplitude; duration and frequency of stimulation) are recorded. Lastly, the total duration of the stimulation period should be noted (e.g., days).

#### 3.3.5 Model‐specific information: TBI

Posttraumatic epilepsy is one of the most commonly reported forms of acquired epilepsy. A wide range of TBI models have been established in adult rodents, with an increasing number of studies exposing mechanisms of epileptogenesis in this context.^41^, ^42^, ^43^ Fewer studies have explored posttraumatic epilepsy in the context of the immature brain, with those conducted to date predominantly using the controlled cortical impact (CCI) model in rats and mice aged P17–P21.^44^, ^45^

This section allows for selection of the TBI model type (CCI)^46^; fluid percussion injury (FPI)^47^; weight drop (WD)^48^ or Marmarou model^49^; blast injury models^50^, ^51^; closed head injury model; penetrating injury, or other. First, general information of importance to all models is requested, including in which experimental group the individual animal is (TBI, sham‐operated, naïve control, or other), where a sham control indicates comparable surgical preparation and procedures to the TBI procedure but no actual impact being delivered. The device used to induce the TBI is specified, as well as the method by which the animal is stabilized to induce the TBI (e.g., stereotaxic frame, restrained, hand‐held). The injury severity (mild, moderate, severe, sub‐concussive, or repetitive) is required, as defined by the investigator, as well as the impact location—cortical region and/or stereotaxic coordinates should be provided. If the injuries are delivered in a repetitive fashion, the number of repeated injuries and time interval between injuries should be reported here. From here, the investigator is able to select to complete “Model‐Specific Parameters” for the appropriate TBI model as selected above (e.g., FPI, CCI, WD). These brief sections are designed to capture pertinent data regarding model‐specific settings such as the angle, tube length, and tube material for the FPI model, as well as the brand of cement used to attach the tubing, and pressure wave pulse and duration settings. For the WD and CCI models, the nature of the impactor tip is essential information, including the surface shape (flat, curved or pointed), composition (metal, plastic, silicone, or other), and diameter; all factors which are known to influence the resulting neuropathology observed.^52^ Details regarding the craniotomy, if performed, is also important to include (e.g., size, shape, and location). For blast injury models, data are sought regarding features of the shock tube, restraint of the animal, and strength of the pressure wave that the animals are exposed to, as these factors are critical determinants of injury severity.^53^

#### 3.3.6 Model‐specific information: Hypoxia and/or ischemia

Perinatal stroke is a common cerebrovascular insult affecting approximately 1 in 4000 live births; and is often associated with the subsequent development of epilepsy.^54^, ^55^ Modeling of perinatal or early postnatal stroke in rodents may be achieved via ligation of the common carotid artery, or photothrombotic lesions (e.g., of the sensorimotor cortex) at around P7.^56^ This method involves injection of a photosensitive dye (e.g., Bengal Rose or Erythrosin B), which is then activated by laser light to induce clot formation leading to ischemic damage. Key parameters to describe include the location of thrombus, details regarding the dye compound used (source, concentration, volume administered, and route), and the nature of the light stimulation (diameter, intensity, and duration). An alternative model of ischemic stroke, the middle cerebral artery occlusion model, is typically performed in slightly older animals (e.g., P10–14).^57^

Similarly, neonatal encephalopathies are often attributed to hypoxia during the perinatal period. Severe neonatal HI brain injury is a leading cause of IS, and can result in epilepsy as well as conditions such as cerebral palsy and intellectual disability.^58^, ^59^ Experimentally, this scenario may be modeled by a combination of ischemic‐reperfusion insult plus a period of hypoxia^60^; or by global hypoxia alone,^61^ typically in rodents aged P7–10. An alternative model involves ligation of the unilateral common carotid artery and subsequent hypoxic challenge, often referred to as the Rice‐Vannucci method.^62^ Critical information to be recorded for these models includes the concentration of oxygen used to induce hypoxia, the concentration of carbon dioxide, timing of seizure onset in relation to hypoxia, as well as the duration of hypoxia and methods used to achieve this.

#### 3.3.7 Model‐specific information: Hyperthermia models

Hyperthermia models are commonly used to recapitulate early life febrile seizures, or convulsions associated with a high body temperature or fever.^63^ While these seizures may be isolated events, and hyperthermia models can also be used to model acute seizures, frequent and/or complex febrile seizures are associated with an increased risk of subsequent epilepsy and adverse consequences in adulthood.^64^, ^65^ Hyperthermia is typically induced in rodents between P7–14, using a heating lamp, mercury vapor lamp, hot water bath, hot air chamber (moist or dry), or other method (to be specified in the CRF). Temperature, duration, and kinetics of the thermal changes induced should be recorded, as well as how the measurements were obtained. Invasive measures include ear, rectal, and thermoregulatory probes, whereas noninvasive methods include biotelemetric devices or thermographic cameras. Models that combine different methods of induction have been created, as for example, hyperthermia with lipopolysaccharide (LPS),^66^ lithium chloride, or pilocarpine.^67^ These can be captured by selecting “mixed model” in the Core CRF (Table 1), under “Models of Epilepsy Syndromes/Seizures” and “Method of induction” and completing both relevant CRFs (“infection‐associated models” section of the Chemical Induction CRF and “Hyperthermia models” CRF module).

#### 3.3.8 Model‐specific information: Cortical freeze models

Physical injury can also be induced by cooling of the neocortical surface, usually achieved by positioning an ultra‐cool probe in contact with the brain tissue. While this procedure results in a focal necrotic lesion resembling a TBI in adult rodents, in the neonatal rodents (up to P4), the same procedure affects the developmental migration of cortical neurons, resulting in disorders such as microgyria or cortical cleft.^68^, ^69^, ^70^ Such models of focal abnormalities of cortical development typically result in brain hyperexcitability by lowering seizure thresholds, and may be combined with secondary insults such as NMDA, flurothyl, pilocarpine, kainic acid, or bicuculline to more closely mimic the clinical phenotype, in which cortical malformations are commonly associated with drug‐resistant epilepsy.^69^ Key information to be noted for the neonatal cortical freeze injury model includes the nature of the device used to induce the injury (composition, size/shape), and the nature of the contact with the brain (duration, temperature, location, depth).

#### 3.3.9 Response to induction model/Procedure

Completing this CRF is a final section to describe the response of the individual animal to the model induction. Duration of apnea, or the latency to resumption of normal breathing, latency to toe pinch response, and latency to self‐righting reflex may all be assessed and considered as surrogate indicators of injury or model severity. Any postprocedure administration of fluids or warming can be detailed. Of particular importance in this context, the observation of any acute postprocedure seizures should be recorded, either briefly within this CRF, or by completing and linking to the more‐detailed *Seizure Phenotyping CRF*. Finally, mortality as a known or suspected consequence of the procedure should be reported.

### 3.4 Specific CRF 2: Chemical induction models (Table 3)

CRF File name: 3. CRF module Chemical induction models.docx

CDE File name: 3. CDE_chart Chemical induction models.xlsx (Supporting information)

**TABLE 3:** Case report form: Specific CRF 2—Chemical induction models of early onset epilepsies and seizures in rodents

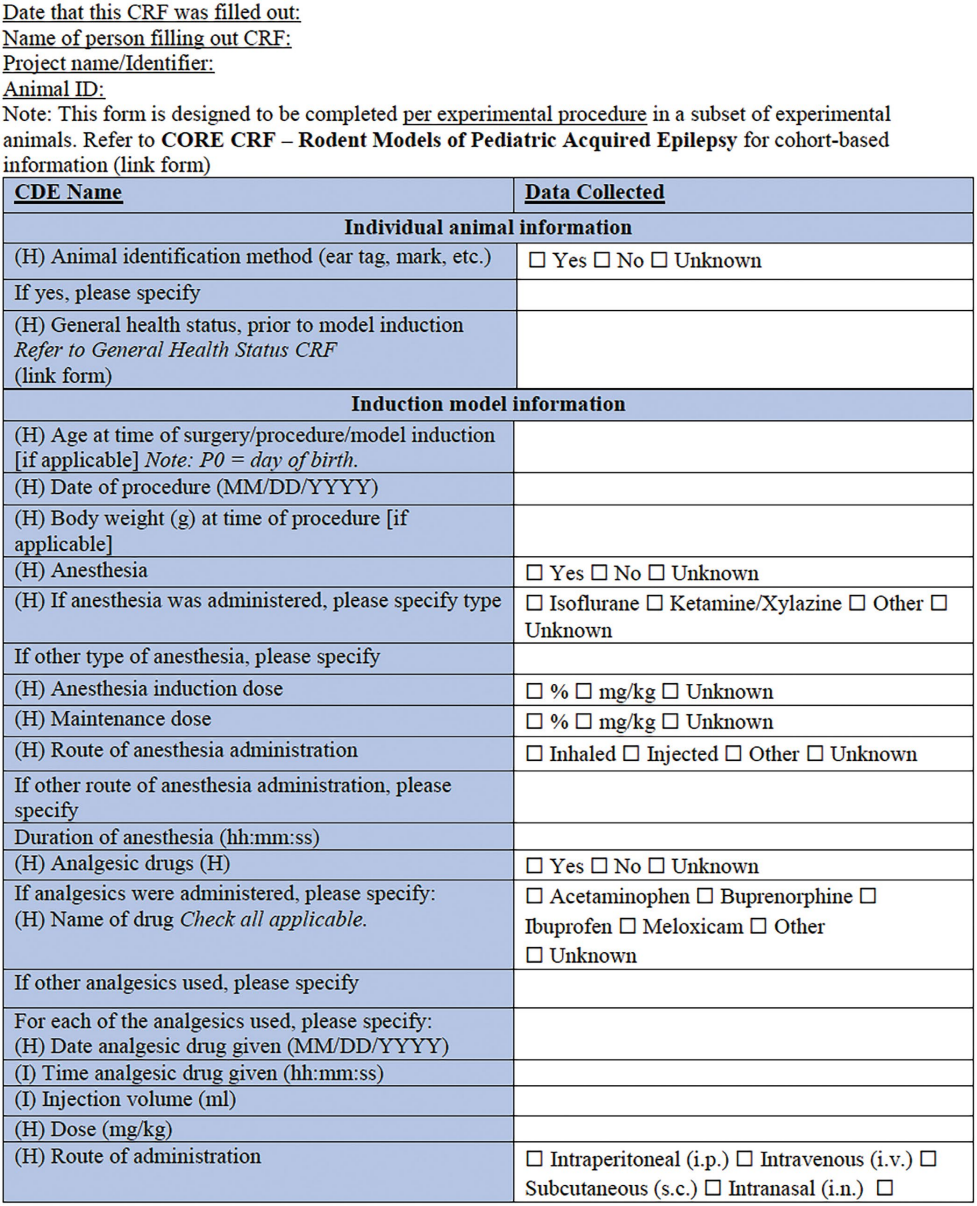
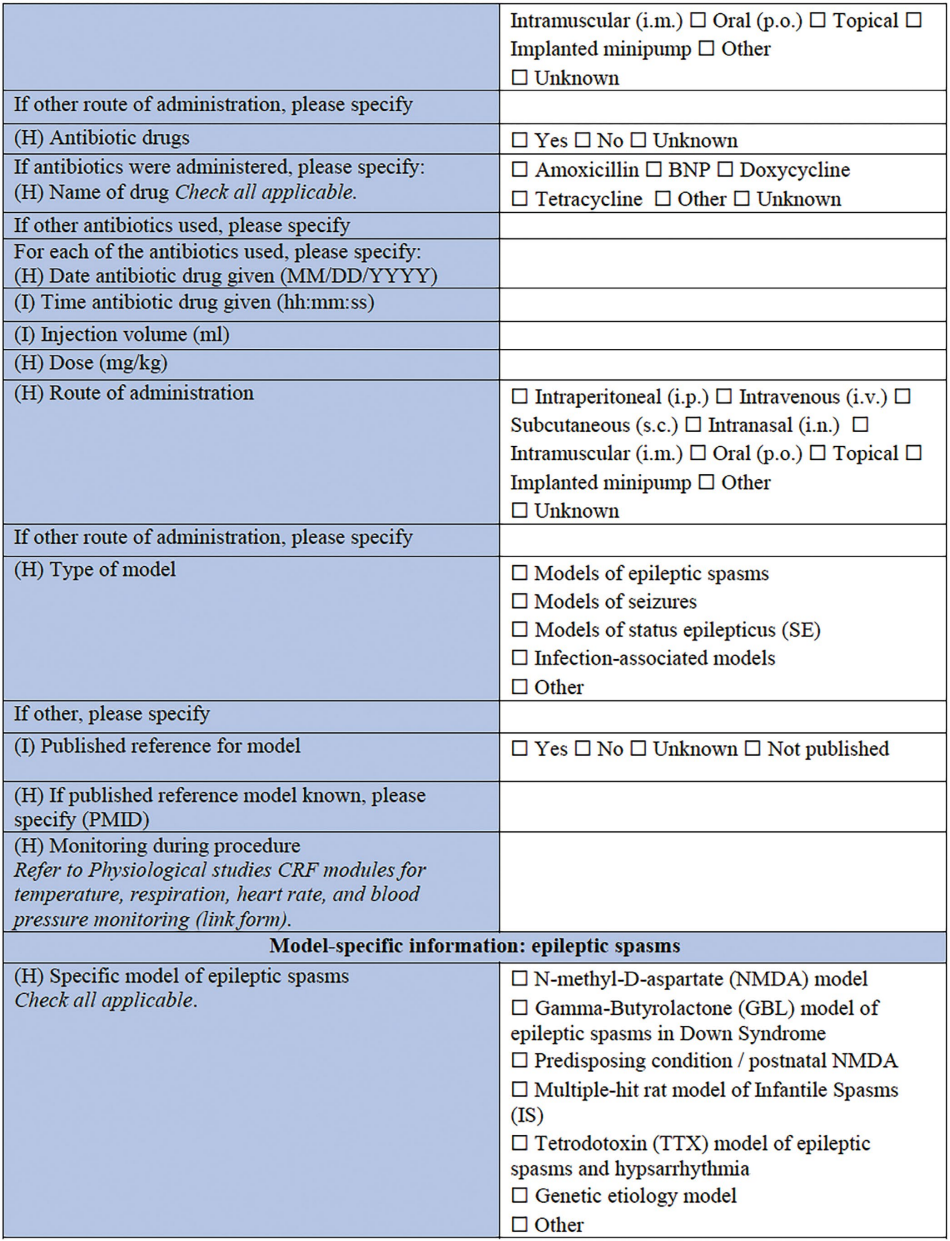
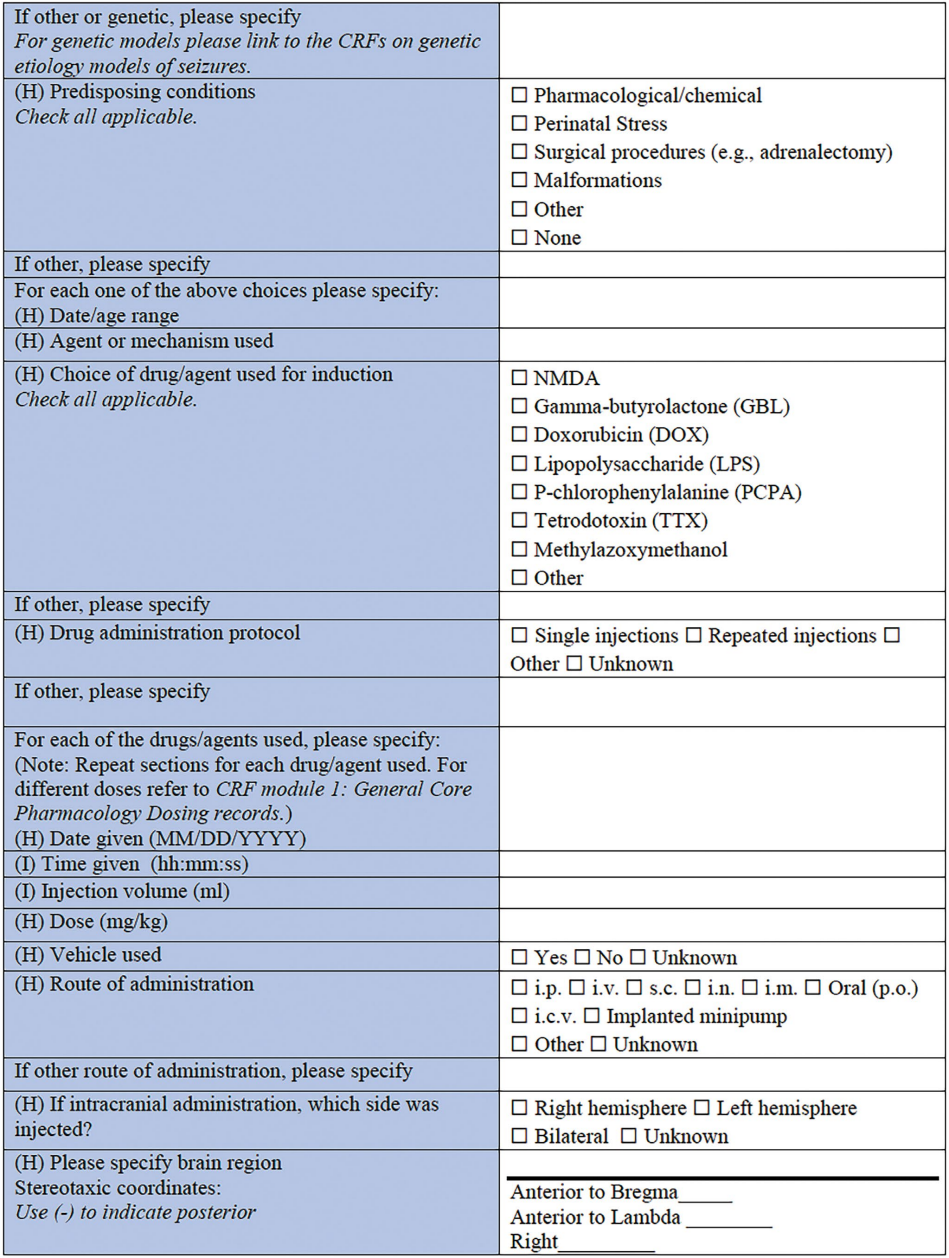
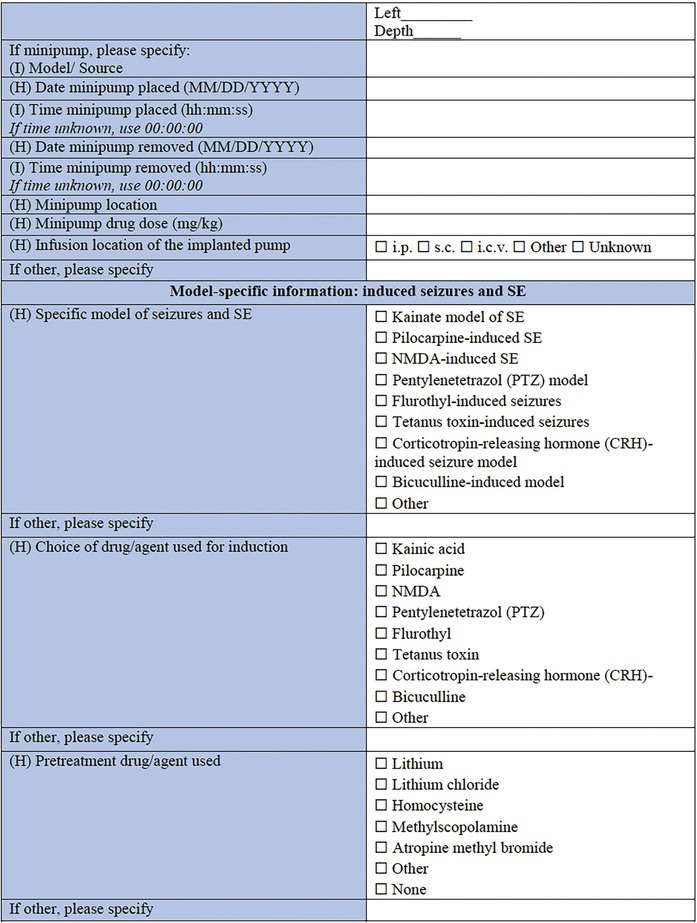
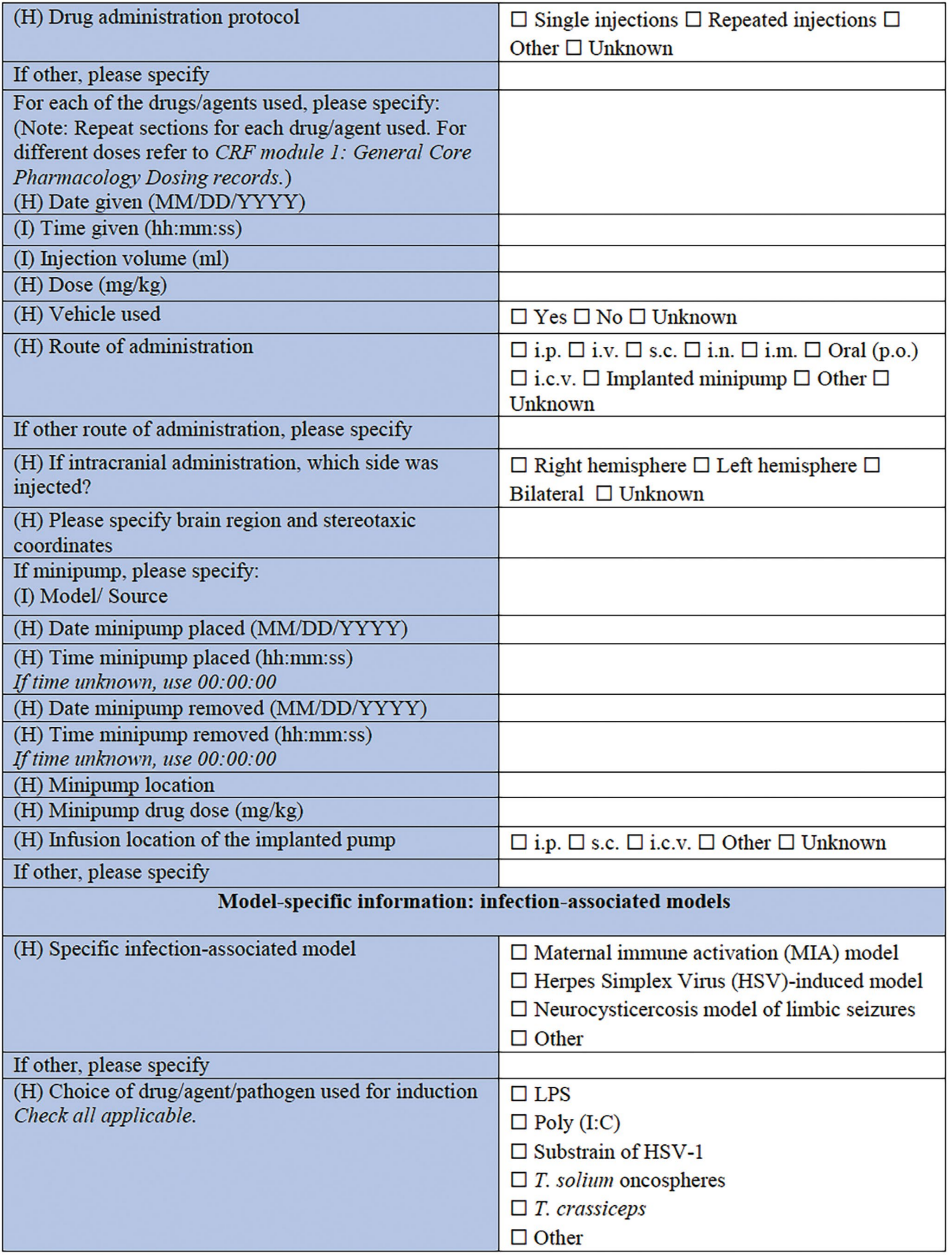
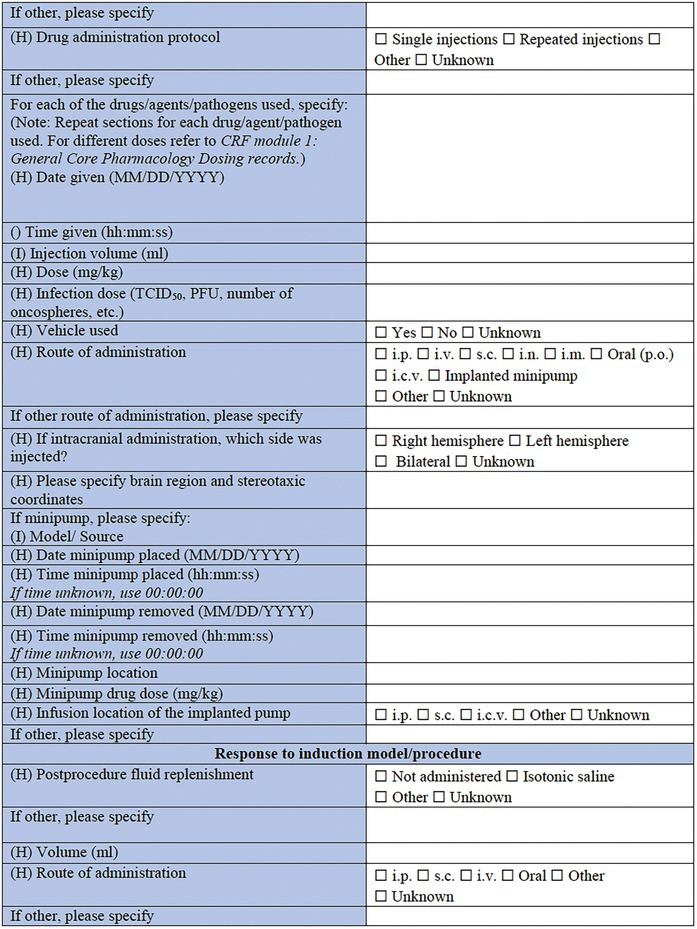
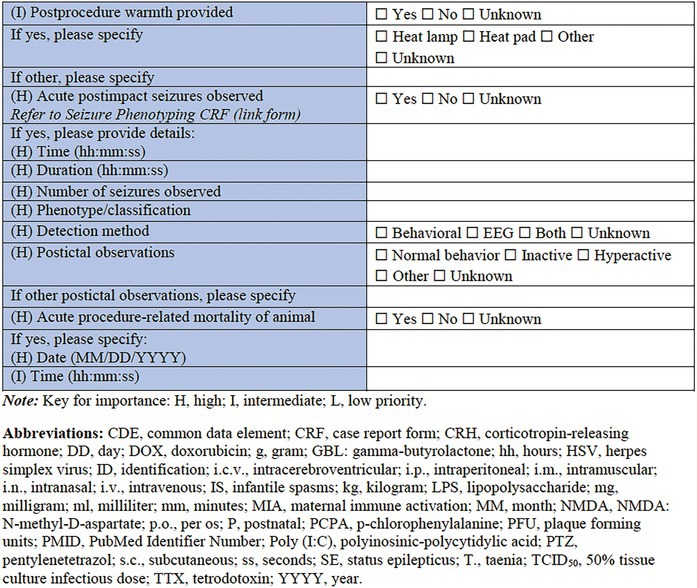

#### 3.4.1 Rationale

This specific CRF is designed to cover rodent models of pediatric acquired epilepsy generated by chemical induction. This category encompasses models that have been described either as acute or chronic models of epileptic spasms; models of SE and seizures provoked using chemoconvulsants; and infection‐associated models based on the administration of chemical components of infectious processes to the immature brain.

#### 3.4.2 Individual animal information

This section provides important information for tracking each animal, such as the animal identification method (ear tag, mark etc.), and the general health status, prior to the model induction, for which a link to the *General Health Status CRF* is also included.

#### 3.4.3 Induction model information

This section records information regarding the specific features of the seizure induction model. It is important to document the type of model, the age, and the body weight of the animal at the time of the procedure, surgery, or model induction, as well as the date that this procedure takes place. If the model induction procedure requires anesthesia, the agent must be specified, as well as details about the anesthesia induction and maintenance dose, the administration route, and the anesthesia duration. If pain medications and antibiotics are used, they should be listed in the relevant sections along with details of the date and time they are administered, the injection volume (mL/kg body weight), the dose (mg/kg), and the route of administration.

In this specific CRF, the categories of chemically induced models that have been included are the models of epileptic spasms, models of SE, and seizures, infection‐associated models, and “other” as an option for models that do not belong in any of those categories. Where possible, a published reference for the chosen model should be listed using the PMID. Monitoring performed during and after the surgical procedure can be documented using the *Physiological Studies CRF* modules (e.g., temperature, respiration, heart rate, and blood pressure monitoring details).^15^

#### 3.4.4 Model‐specific information: Epileptic spasms

Several acute or chronic models of epileptic spasms or IS have been developed so far, due to the increased need to find new therapeutic pathways for these rare but severe developmental and epileptic encephalopathies. The specific models of epileptic spasms listed in this CRF are the following: the N‐methyl‐d‐aspartate (NMDA) model^71^, ^72^, ^73^, ^74^, ^75^; the gamma‐butyrolactone (GBL) model of epileptic spasms in Down Syndrome^76^; the prenatal betamethasone/postnatal NMDA model and other NMDA models in animals with predisposing conditions^72^, ^77^, ^78^; the multiple‐hit rat model of infantile spasms^79^; and the tetrodotoxin (TTX) model of epileptic spasms and hypsarrhythmia.^80^ “Other” is an available choice in case this CRF is used for a model that is not listed here.

The predisposing conditions include pharmacological/chemical (i.e., betamethasone, methylazoxymethanole), stress, surgical procedures such as adrenalectomy, or any malformations (as after prenatal methylazoxymethanole administration) or specific genetic background,^81^ that could have a predisposing effect on the model induction. Details referring to the date and age range pertinent to the predisposing conditions should also be recorded in this section.

A list of choices of drugs and agents that are used for the induction of the above‐mentioned models comes next. This list includes NMDA, GBL, doxorubicin (DOX), lipopolysaccharide (LPS), p‐chlorophenylalanine (PCPA), TTX, and “other” for additional options.

Drug administration protocol information should then be provided, as well as specific details for each of the drugs/agents used—the date and the time of administration, the injection volume (mL/kg body weight), and the dose (mg/kg), the usage (or not) of a vehicle, and the administration route. This section can be repeated for each of the different agents required for a particular model. For the report of different doses, the *CRF module 1: General Core Pharmacology Dosing records* by TASK3‐WG1A General Pharmacology Working Group of the ILAE/AES Joint Translational Task Force can be used. If the drug or agent administration is intracranial, the side of the brain should be noted (right hemisphere, left hemisphere, or bilateral). The brain region and the coordinates relative to anatomic landmarks that are used for the stereotaxic infusions of the pharmacologic compounds should also be provided. In case the administration method contains minipump implantation, additional data should be collected, such as the model or commercial source of the minipump, the date and the time of minipump placement and subsequent removal, pump location, the dose of the pharmacologic/chemical agent included (mg/kg), and the infusion location of the implanted pump (i.p., s.c., i.c.v., other).

#### 3.4.5 Model‐specific information: Induced seizures and SE

A great variety of seizure models and SE models have been developed over the years in the immature brain, to investigate the higher incidence of seizures observed in infants, as well as define the differences in SE between infants and adults in humans.^64^, ^82^, ^83^ Specific models of induced seizures and SE in this section include the kainate model of SE,^84^, ^85^, ^86^ the pilocarpine‐induced SE,^87^, ^88^, ^89^ the NMDA‐induced SE,^71^, ^74^, ^75^ the pentylenetetrazol (PTZ) model,^32^, ^90^, ^91^, ^92^ and flurothyl‐induced seizures.^93^ The choice of drugs and/or agents used for induction follows, which in this case includes kainic acid, pilocarpine, NMDA, PTZ, flurothyl, tetanus toxin,^94^ corticotropin‐releasing hormone (CRH),^95^ bicuculline,^96^ and other (to be specified). Next, pretreatment drugs/agents used as part of the protocol of the previously mentioned models should be reported, such as lithium chloride, homocysteine,^97^ methylscopolamine, and atropine methyl bromide.^98^ The sections that follow are similar to the ones used for the models of epileptic spasms: the drug administration protocol section, the specific information for each of the drugs/agents used section, the side of the brain along with the stereotaxic coordinate details needed in case of intracranial administration, and the minipump information that need to be reported in case the model requires minipump implantation.

#### 3.4.6 Model‐specific information: Infection‐associated models

A wide range of infectious agents have been associated with seizures and epilepsy.^99^, ^100^ That observation led to the description of several infection‐induced animal models, which allow for exploration of the impact that neuropathological and inflammatory changes may have to the immature brain.^30^ The list of specific infection‐associated models includes the maternal immune activation (MIA) model,^101^, ^102^ the herpes simplex virus (HSV)‐induced model,^103^, ^104^, ^105^, ^106^, ^107^, ^108^ and the neurocysticercosis model of limbic seizures.^109^, ^110^ “Other” is an available choice in this subsection too, in case this CRF is used for a model that is not listed here. What follows is the choice of drug, agent, as well as pathogen used for the induction of this type of model. This section includes LPS, polyinosinic‐polycytidylic acid [Poly (I:C)], a sub‐strain of HSV‐1, *Taenia solium* oncospheres, and *Taenia crassiceps* as some of the available options. This is not to say that other models are not valid; but these selected models have an extensive history and detailed protocols have been well described across multiple investigative teams. The sections about administration protocol, specific information for each of the components used, intracranial administration, and details about minipumps implantation are listed here too. The only addition in this section is the infection dose, including the options of the 50% tissue culture infectious dose (TCID_50_), the plaque forming units (PFU), and the number of oncospheres, as part of the dose information, where pathogens are used for model induction. This section may also be relevant for combined physical and chemical induction models, such as hyperthermia in addition to LPS.^66^

#### 3.4.7 Response to induction model/Procedure

This is the final section of the Specific CRF used to describe the response of the individual animal to the induction model. Any postprocedure administration of fluids or warming can be reported in detail here. It is critically important in this section to record the observation of any acute postprocedure seizures. This should be recorded either briefly within this CRF, or by using the more detailed linked *Seizure Phenotyping CRF*. Last, but not least, acute procedure‐related mortality of the animal should be reported.

## 4 DISCUSSION

Early onset or childhood epilepsy syndromes are associated with a high risk for considerable neurocognitive comorbidities and poor psychosocial outcomes, which can negatively impact quality of life for the affected individuals and their family.^111^ There is thus a need for improved treatment options to target early life epilepsy syndromes, considering specific seizure phenotypes and age‐specific effects. Preclinical work utilizing animal models can develop the foundation for such advancements, by providing mechanistic insight into the epileptogenic process. Animal models aim to recapitulate both the triggering insult of acquired epilepsy (e.g., TBI, stroke, SE, or infection) as well as the resulting seizure phenotypes, and feature many of the disease phenotypes of patients with early life epilepsy. Such models are imperative to our advancement in understanding the fundamental neurobiology that underlies acquired epilepsies. Further, preclinical models of acquired epilepsy allow for investigation of the impact of genetic predisposition and comorbidities, and the testing of novel therapeutic interventions. Standardization of these models, and transparency of reporting of model induction parameters, is imperative for optimal scientific rigor and reproducibility, and suggestions for improvement, recommendations, or guidelines have been published by several groups.^7^, ^112^, ^113^, ^114^, ^115^, ^116^ Beyond its paramount role in promoting transparency and comparisons of experimental data, standardization of experimental approaches may minimize variability stemming from different experimental conditions, including but not limited in handling methods, stressors, diet, and housing. Recommendations and guidance to improve conditions of experimentation, particularly for seizure models, have been published with the intent to promote and advocate for animal welfare and humane experimentation.^116^, ^117^, ^118^ These include both local regulatory bodies for animal experimentation as well as international efforts to improve animal welfare in experimental studies, such as the ARRIVE guidelines.^112^, ^116^

In this companion manuscript, we have described newly generated CRFs and CDEs designed to fill this knowledge gap. The *Core CRF* provides preclinical researchers with a means to define the key characteristics of a specific cohort of experimental animals. This is to be used in conjunction with one of two *Specific CRFs*, to describe the specific physical or chemical induction model that is being used (Figure 1). Together, these forms can be complemented with existing CRFs already available in both hard‐copy and electronic format (EPICDE@LONI; https://ida.loni.usc.edu/home/projectPage.jsp?project=ILAE), providing an opportunity to readily integrate data collection, management, and storage in a structured and logical manner. The electronic CRFs may also be downloaded from the “Preclinical epilepsy common data elements (EPICDE)” page of the ILAE website: https://www.ilae.org/about‐ilae/topical‐commissions/yes/commission‐on‐neurobiology/resources. These downloadable, editable documents are intended to be printed and manually filled or completed electronically, for practical implementation in laboratories worldwide, as detailed previously.^7^

As with all CRFs and CDEs to date, there will be some challenges and limitations associated with the use of these documents. The importance of diligently inputting detailed and complete information cannot be understated, ensuring that the resulting data sets are as accurate as possible. We have endeavored to include in the CRFs the most pertinent information to be recorded for each model, although many factors specific to a given model may not be covered. We encourage experts in any given model to invest the time to describe their model in sufficient detail, as well as the importance of different characteristics and features of the model. Finally, we aimed to balance between providing comprehensive coverage of existing models and ensuring a manageable scope such that the resulting documents are both user‐friendly and practical. As a result, not all possible models of early life onset epilepsy syndromes have been included. For example, brain tumors may be responsible for some childhood epilepsies, and a greater understanding of how glioma‐induced epileptogenesis is necessary in both the adult and immature brain.^119^ Epilepsies that develop as consequences of congenital brain malformations of prenatal origin, or birth‐related trauma, have also largely been excluded—although models of the former conditions may be captured via the new *Genetic CRF modules* where known genetic mutations are modeled to recapitulate the disease.

## 5 CONCLUSION

In summary, we have developed a Core CRF for the reporting of rodent models of pediatric acquired epilepsies, to be used alongside one of two Specific CRFs for physical or chemical induction models, respectively. These CRFs provide investigators with the tools to systematically record critical information regarding their chosen experimental model, for improved standardization and transparency across laboratories. This companion manuscript describes the importance of each element of the CRFs, highlighting the potential impact of each aspect on the resulting phenotype of the model. Use of these documents to enhance scientific rigor and reproducibility in preclinical models of pediatric epilepsy syndromes is anticipated to drive the field toward improved understanding of fundamental neurobiology alongside novel therapeutic discovery of relevance to acquired pediatric epilepsies.

## CONFLICT OF INTEREST

AS Galanopoulou is Editor‐in‐Chief of *Epilepsia Open* and Associate Editor of *Neurobiology of Disease* and has received royalties for publications from Elsevier, Medlink, and Morgan and Claypool publishers. None of the other authors have conflicts to disclose.

## ETHICS STATEMENT

All authors confirm that we have read the Journal's position on issues involved in ethical publication and affirm that this report is consistent with those guidelines. Investigators who wish to access the existing electronic CRFs of the TASK3 may download the files from the “Preclinical epilepsy common data elements (EPICDE)” page of the ILAE website: https://www.ilae.org/about‐ilae/topical‐commissions/yes/commission‐on‐neurobiology/resources.

## Supporting information


Appendix S1
