# Supplementary material for: A companion to the preclinical common data elements for rodent models of pediatric acquired epilepsy: A report of the TASK3‐WG1B, Pediatric and Genetic Models Working Group of the ILAE/AES Joint Translational Task Force
Source: Epilepsia Open. 2022 Oct 5;10(Suppl 1):S53–86. doi: 10.1002/epi4.12641 (PMC12375983; doi:10.1002/epi4.12641)
Supplement: Supplementary file 1 — Appendix S1 [file EPI4-10-S53-s001.zip › EPI4_12641_1. Core CRF module Rodent models of pediatric acquired epilepsy.docx]

**Table 1:**

**Case Report Form: CORE CRF – Rodent Models of Pediatric Acquired Epilepsy**

Date that this CRF was filled out:

Name of person filling out CRF:

Project name/Identifier:

Animal ID:

Note: This form is designed to be completed per cohort of experimental animals.

Refer to Specific CRFs on *Physical induction models* or *Chemical induction models* for individual animal information.

| **CDE Name** | **Data Collected** |
| --- | --- |
| **General animal information** | |
| (H) Species | ☐ Mouse ☐ Rat ☐ Other |
| If other species type, please specify |  |
| (H) If mouse, what strain? | ☐ C57Bl/6 ☐ S129V ☐ A/J ☐ DBA  ☐ BALB/C ☐ S129V ☐ CD-1 ☐ SCID ☐ Other  ☐ Unknown |
| If other mouse strain, please specify |  |
| (I) If sub-strain (e.g. C57BL/6J or 6N) known, please specify |  |
| (H) If rat, what strain? *Check all applicable for mixed breed.* | ☐ Wistar ☐ Sprague-Dawley ☐ Long-Evans  ☐ Mixed ☐ Other ☐ Unknown |
| If other type of strain, please specify |  |
| (H) Animal source/vendor |  |
| (H) Biological sex | ☐ Male ☐ Female ☐ Unknown |
| **Genetic modification** | |
| (H) Genetic modification | ☐ Yes ☐ No ☐ Unknown |
| (H) Please indicate type of genetic modification  *To record method, type, expression refer to Genetic Rodent Models CRF* (link to genetic models CRF.docx). | ☐ Genetic; engineered  ☐ Genetic; spontaneous (naturally-occurring) |
| **Pre-weaning housing** | |
| (I) Pathogen-free housing | ☐ Yes ☐ No ☐ Unknown |
| (H) Litter name/number [if applicable] |  |
| (I) Parent’s ID male [if applicable] |  |
| (I) Parent’s ID female [if applicable] |  |
| (H) Date of birth: is it known? | ☐ Yes ☐ No |
| If the DOB is known, please specify (MM/DD/YYYY)  *Note: If exact date is not known, but the week is known, enter Monday of that week.* |  |
| **Litter characteristics at first observation** | |
| (H) Date (MM/DD/YYYY) |  |
| (I) Number of pups born |  |
| (H) Number of live pups |  |
| (H) If weaned, at what age |  |
| (I) Ratio in the litter male: female pups |  |
| (H) Are the pups fostered? | ☐ Yes ☐ No ☐ Unknown |
| **Post-weaning housing** | |
| (I) Pathogen-free housing | ☐ Yes ☐ No ☐ Unknown |
| **Litter characteristics at observation** | |
| (H) Date (MM/DD/YYYY) |  |
| (H) Number of live pups |  |
| (I) Ratio in the litter male: female pups |  |
| (H) Detailed housing and environmental conditions (vivarium)  *Refer to Core Animal Characteristics CRF* (link to Core CRF.docx). |  |
| **Experimental housing** | |
| (H) Animals kept in what kind of facility | ☐ Animal facilities ☐ Laboratory room ☐ Other ☐ Unknown |
| If other, please specify |  |
| (H) Animal housing chamber | ☐ Cage ☐ Beaker/cylinder ☐ Box  ☐ Other ☐ Unknown |
| If other, please specify |  |
| (L) Dimensions (e.g., of cage, beaker/ cylinder, etc.) in cm, if known | W___ x L___ x H___  or __________ diameter x H ___ |
| (I) Bedding | ☐ Yes ☐ No ☐ Unknown |
| (L) If bedding was present, please specify | ☐ Corncob ☐ Aspen wood ☐ Paper ☐ Other  ☐ Unknown |
| If other type of bedding, please specify |  |
| (I) Enrichment? | ☐ Yes ☐ No ☐ Unknown |
| (I) If enrichment was present, describe |  |
| (H) Room temperature range in (^o^C) | ________ (low) - _________ (high) |
| (H) Relative humidity range (%) | ________ (low) - __________ (high) |
| (H) Light-dark cycle type | ☐ Normal ☐ Reversed ☐ Other ☐ Unknown |
| (H) Type of light  *Check all applicable.* | ☐ Natural light ☐ Infrared light ☐ Other ☐ Unknown |
| If other type of light, please specify |  |
| (I) Lights switched on (hh:mm) |  |
| (I) Lights switched off (hh:mm) |  |
| (I) Instantaneous change in lighting | ☐ Yes ☐ No ☐ Unknown |
| If no, describe light settings |  |
| (H) Animals separated during monitoring | ☐ Yes ☐ No ☐ Unknown |
| If no, specify number of animals per cage |  |
| (I) Type of food: if known, specify | ☐ Pellet ☐ Milk ☐ Other |
| If other type of food, please specify |  |
| (H) Access to food | ☐ Ad libitum ☐ Restricted ☐ Unknown |
| (L) If restricted, please specify | ☐ Caloric restriction ☐ Intermittent feeding ☐ Unknown ☐ Other |
| If access to food was other, please specify |  |
| (I) Type of water | ☐ Tap water ☐ Autoclaved ☐ Other ☐ Unknown |
| If other type of water, please specify |  |
| (H) Access to water | ☐ Ad libitum ☐ Other ☐ Unknown |
| If other type of access to water, please specify |  |
| **Models of Epilepsy Syndromes/Seizures** | |
| (H) Method of induction  *If chemical: refer to Specific CRF – Chemical induced models of early onset epilepsies and seizures in rodents (link form).*  *If physical: refer to Specific CRF – Physical induction models of early onset epilepsies and seizures in rodents (link form).*  *If mixed; (refer to all-appropriate Specific CRF/s).* | ☐ Chemical (e.g., NMDA, GBL or TTX administration)  ☐ Physical (e.g., trauma/stroke model; electrical stimulation)  ☐ Mixed  ☐ Other  ☐ Unknown |
| If other method of induction, please specify *(Refer to most-appropriate Specific CRF).* |  |
| (H) Date of model induction (MM/DD/YYYY) |  |
| **Measurements and Outcomes** | |
| (H) Behavioral developmental milestones measured?  *Refer to Developmental Milestones CRF (link form).* | ☐ Yes ☐ No ☐ Unknown |
| (H) Behavioral tests conducted  *Refer to appropriate Neurobehavioral Comorbidities CRF Modules (link form).* | ☐ Yes ☐ No ☐ Unknown |
| (H) Specify seizure phenotype  *Refer to CRF Module for Seizure Phenotyping (link form).* |  |
| (H) EEG information  *Refer to CRF Modules for recording, analyzing, and scoring preclinical EEG (Ono et al., 2018), and methodological recommendations for video-EEG recordings in immature rodents (Akman et al., 2018) (link forms).* | ☐ Yes ☐ No ☐ Unknown |
| (H) Video recording and analysis  *Refer to CRF Modules for recording, analyzing, and scoring preclinical EEG (Ono et al., 2018), and methodological recommendations for video-EEG recordings in immature rodents (Akman et al., 2018) link forms).* | ☐ Yes ☐ No ☐ Unknown |
| (H) Terminal time point – Euthanasia | ☐ Yes ☐ No ☐ Unknown |
| (H) Method of euthanasia  *Check all applicable.* | ☐ Chemical ☐ Physical ☐ Other ☐ Unknown |
| If other, please specify |  |
| (H) If chemical method of euthanasia, please specify | ☐ Carbon dioxide (CO2) ☐ Ketamine/Xylazine ☐ Pentobarbital ☐ Phenobarbital ☐ Other  ☐ Unknown |
| If other, please specify |  |
| (H) If physical method of euthanasia, please specify | ☐ Cervical dislocation ☐ Decapitation  ☐ Transcardial perfusion ☐ Other ☐ Unknown |
| If other, please specify |  |
| (H) If anesthesia used, please specify | ☐ Isoflurane ☐ Sevoflurane  ☐ Ketamine/Xylazine ☐ Other  ☐ Unknown |
| If other, please specify |  |
| (H) Date of euthanasia (MM/DD/YYYY) |  |
| (L) Time of euthanasia (Hh:mm:ss) |  |
| (H) Tissue collection | ☐ Yes ☐ No ☐ Unknown |
| (H) If tissue was collected, was neuropathological evaluated  *Refer to CRFs for Neuropathological evaluation studies in rodent epilepsy models*  (link forms). | ☐ Yes ☐ No ☐ Unknown |

**Abbreviations:** CRF: case report form; ID: identification; CDE: common data element; DOB: date of birth; MM: month; DD: day; YYYY: year; W: width; L: length; H: height; cm: centimeter; hh: hours; mm: minutes; NMDA: N-methyl-D-aspartate; GBL: Gamma-Butyrolactone; TTX: Tetrodotoxin; EEG: electroencephalogram; CO2: carbon dioxide. Key for importance: H = high, I = intermediate and L = low priority.
