# Supplementary material for: A companion to the preclinical common data elements for rodent models of pediatric acquired epilepsy: A report of the TASK3‐WG1B, Pediatric and Genetic Models Working Group of the ILAE/AES Joint Translational Task Force
Source: Epilepsia Open. 2022 Oct 5;10(Suppl 1):S53–86. doi: 10.1002/epi4.12641 (PMC12375983; doi:10.1002/epi4.12641)
Supplement: Supplementary file 1 — Appendix S1 [file EPI4-10-S53-s001.zip › EPI4_12641_2. CRF module Physical induction models.docx]

**Table 2:**

**Case Report Form:**

**Specific CRF 1– Physical induction models of early onset epilepsies and seizures in rodents**

Date that this CRF was filled out:

Name of person filling out CRF:

Project name/Identifier:

Animal ID:

Note: This form is designed to be completed per experimental procedure in a subset of experimental animals. Refer to **CORE CRF – Rodent Models of Pediatric Acquired Epilepsy** for cohort-based information (link form)

| **CDE Name** | **Data Collected** |
| --- | --- |

| **Individual animal information** | |
| --- | --- |
| (H) Animal identification method (ear tag, mark, etc.) | ☐ Yes ☐ No ☐ Unknown |
| If yes, please specify |  |
| (H) General health status, prior to model induction  *Refer to General Health Status CRF*  (link form) |  |

| **Induction model information** | |
| --- | --- |
| (H) Age at time of surgery/procedure/model induction [if applicable] *Note: P0 = day of birth.* |  |
| (H) Date of procedure (MM/DD/YYYY) |  |
| (H) Body weight (g) at time of procedure [if applicable] |  |
| (H) Anesthesia | ☐ Yes ☐ No ☐ Unknown |
| If anesthesia was administered, please specify type | ☐ Isoflurane ☐ Ketamine/xylazine  ☐ Other ☐ Unknown |
| If other type of anesthesia, please specify |  |
| (H) Anesthesia induction dose | ☐ % ☐ mg/kg ☐ Unknown |
| (H) Maintenance dose | ☐ % ☐ mg/kg ☐ Unknown |
| (H) Route of anesthesia administration | ☐ Inhaled ☐ Injected ☐ Other ☐ Unknown |
| If other route of anesthesia administration, please specify |  |
| (H) Duration of anesthesia (hh:mm:ss) |  |
| (H) Analgesic drugs | ☐ Yes ☐ No ☐ Unknown |
| If analgesics were administered, please specify:  (H) Name of drug *Check all applicable.* | ☐ Acetaminophen ☐ Buprenorphine ☐ Ibuprofen ☐ Meloxicam ☐ Other  ☐ Unknown |
| If other analgesics used, please specify |  |
| For each of the analgesics used, please specify:  (H) Date analgesic drug given (MM/DD/YYYY) |  |
| (I) Time analgesic drug given (hh:mm:ss) |  |
| (I) Injection volume (mL) |  |
| (H) Dose (mg/kg) |  |
| (H) Route of administration | ☐ Intraperitoneal (i.p.) ☐ Intravenous (i.v.) ☐ Subcutaneous (s.c.) ☐ Intranasal (i.n.) ☐ Intramuscular (i.m.) ☐ Oral (p.o.)  ☐ Other ☐ Unknown |
| If other route of administration, please specify |  |
| (H) Antibiotic drugs | ☐ Yes ☐ No ☐ Unknown |
| If antibiotics were administered, please specify:  (H) Name of drug *Check all applicable.* | ☐ Amoxicillin ☐ BNP ☐ Doxycycline ☐ Tetracycline ☐ Other ☐ Unknown |
| If other antibiotics used, please specify |  |
| For each of the antibiotics used, please specify:  (H) Date antibiotic drug given (MM/DD/YYYY) |  |
| (I) Time antibiotic drug given (hh:mm:ss) |  |
| (I) Injection volume (mL) |  |
| (H) Dose (mg/kg) |  |
| (H) Route of administration | ☐ Intraperitoneal (i.p.) ☐ Intravenous (i.v.) ☐ Subcutaneous (s.c.) ☐ Intranasal (i.n.) ☐ Intramuscular (i.m.) ☐ Oral (p.o.)  ☐ Other ☐ Unknown |
| If other route of administration, please specify |  |
| (H) Type of model | ☐ Electrical stimulation-induced seizures  ☐ Traumatic brain injury (TBI) models  ☐ Ischemic stroke model  ☐ Hypoxic-ischemic (HI) injury  ☐ Hypoxia-induced seizures  ☐ Hyperthermia models  ☐ Cortical freeze injury  ☐ Other |
| If other, please specify |  |
| (I) Published reference for model | ☐ Yes ☐ No ☐ Unknown ☐ Not published |
| (I) If published reference model known, please specify (PMID) |  |
| (H) Monitoring during procedure  *Refer to Physiological studies CRF modules for temperature, respiration, heart rate, and blood pressure monitoring (link form).* |  |
| **Model-specific information: Electrical stimulation-induced seizures** | |
| (H) Model | ☐ Kindling  ☐ Perforant path stimulation (PP)  ☐ Maximal electroshock seizure  ☐ 6Hz electrical stimulation  ☐ Transauricular kindling  ☐ Corneal stimulation  ☐ Other |
| If other, please specify |  |
| (H) Site of electrical stimulation | ☐ Amygdala  ☐ Hippocampus  ☐ Cerebral cortex  ☐ Corneal  ☐ Transauricular  ☐ Perforant pathway  ☐ Other  ☐ Unknown |
| If other location, or exact location needs specification, please specify |  |
| (H) Laterality of stimulation | ☐ Left hemisphere ☐ Right hemisphere  ☐ Bilateral ☐ Unknown |
| **Electrodes** | |
| (H) Material [if applicable] |  |
| (H) Electrode type / model:   - (For specific CRFs on electrodes, see PMID: **30450486)** |  |
| (H) Source/ Vendor (if not homemade) |  |
| (H) Method of electrode placement |  |
| (H) Location (Brain area / stereotaxic coordinates) |  |
| (H) Verification of electrode placement | ☐ Yes ☐ No ☐ Unknown |
| **Parameters of stimulation** | |
| (H) Trains | ☐ Unilateral ☐ Bilateral ☐ Unknown |
| (H) Pulses | ☐ Monophasic ☐ Biphasic ☐ Unknown |
| (H) Pulse Frequency (Hz) |  |
| (H) Current (µA) |  |
| (I) Ramp Current (µA), range (optional, if done) | Min_____; Max_____ |
| (H) Duration of individual stimulation events (hr:min:sec) |  |
| (H) Frequency of stimulations per day  (optional, if repeated stimulation done) | ☐ Once  ☐ Monophasic |
| (H) Number of stimulations |  |
| (H) Frequency of stimulations per day  (*optional, if number of stimulations >1/day*) |  |
| (H) Duration of stimulation period (hr:min:sec) |  |
| *Filename of uploaded relevant protocol or published reference, if available. (Optional)* |  |
| **Model-specific information: Traumatic brain injury (TBI)** | |
| (H) Injury model | ☐ Controlled cortical impact (CCI)  ☐ Fluid percussion injury (FPI)  ☐ Weight drop (WD) or Marmarou model  ☐ Blast injury  ☐ Closed head injury (CHI)  ☐ Other |
| If other, please specify |  |
| (H) Injury group | ☐ TBI ☐ Sham-operated ☐ Naïve control  ☐ Other |
| If other, please specify |  |
| (I) Device manufacturer |  |
| (L) Date of last calibration/service [if applicable] (MM/DD/YYYY) |  |
| (H) Animal stabilization method | ☐ Stereotaxic frame ☐ Hand-held  ☐ Restrained ☐ Other ☐ Unknown |
| If other stabilization method, please specify |  |
| (H) Scalp incision | ☐ Yes ☐ No ☐ Unknown |
| If yes, please specify method of incision closure post-operative | ☐ Suture ☐ Glue ☐ Staple ☐ Not closed ☐ Unknown |
| (H) Craniotomy | ☐ Yes ☐ No ☐ Unknown |
| If yes, please specify:  (I) Drill bit diameter used (mm) |  |
| (H) Craniotomy diameter (mm) |  |
| (H) Damage to dura | ☐ Yes ☐ No ☐ Unknown |
| (H) Impact location side | ☐ Left ☐ Right ☐ Midline ☐ Other  ☐ Unknown |
| (H) Impact location cortical region | ☐ Frontal ☐ Parietal ☐ Other ☐ Unknown |
| (I) Coordinates [if applicable] |  |
| (H) Injury severity | ☐ Mild (including concussion) ☐ Moderate ☐ Severe ☐ Sub-concussive  ☐ Repetitive ☐ Other |
| If other, please specify |  |
| (H) Repeated induction | ☐ Yes ☐ No ☐ Unknown |
| If repetitive, please specify:  (H) How many |  |
| (H) What interval |  |
| (H) Order of this procedure |  |
| **MODEL-SPECIFIC PARAMETERS: CCI** | |
| (I) Device (vendor, make, model) |  |
| (H) Impactor tip: Diameter (mm) [if applicable] |  |
| (H) Impactor surface | ☐ Flat ☐ Curved ☐ Point ☐ Unknown |
| (I) Impactor composition | ☐ Metal ☐ Plastic ☐ Silicone ☐ Other ☐ Unknown |
| If other composition, please specify |  |
| (H) Impactor angle (°) [if applicable] |  |
| (H) Impact depth (mm) [if applicable] |  |
| (I) Impact duration/dwell time (ms) [if applicable] |  |
| (I) Impact velocity (m/s) [if applicable] |  |
| **MODEL-SPECIFIC PARAMETERS: FPI** | |
| (I) Device (vendor, make, model) |  |
| (H) Connector angle (°) [if applicable] |  |
| (H) Connector tube: Length (mm) [if applicable] |  |
| (I) Connector tube: Material [if applicable] |  |
| (L) Cement brand [if applicable] |  |
| (H) Pressure wave: Pulse (atm) [if applicable] |  |
| (I) Pressure wave: Duration (mm:ss) [if applicable] |  |
| **MODEL-SPECIFIC PARAMETERS: WD** | |
| (I) Device (vendor, make, model) |  |
| (H) Impactor tip: Diameter (mm) [if applicable] |  |
| (H) Impactor surface | ☐ Flat ☐ Curved ☐ Point ☐ Unknown |
| (I) Impactor composition | ☐ Metal ☐ Plastic ☐ Silicone ☐ Other  ☐ Unknown |
| If other composition, please specify |  |
| (H) Impactor rod: Length (mm) [if applicable] |  |
| (H) Impactor rod: Mass (g) [if applicable] |  |
| (I) Impactor rod: Material [if applicable] |  |
| (H) Weight drop: Height (m:cm:mm) [if applicable] |  |
| (H) Weight drop: Guidance | ☐ Unguided (free-fall) ☐ Within tube  ☐ Other ☐ Unknown |
| If other guidance, please specify |  |
| (I) Impact duration/dwell time (ms) [if applicable] |  |
| (I) Impact velocity (m/s) [if applicable] |  |
| (I) Rod retraction post-impact | ☐ Yes ☐ No ☐ Unknown |
| If yes, please specify method | ☐ Computer-driven ☐ Manual  ☐ Unknown |
| (I) Contact surface: Material | ☐ Metal ☐ Plastic ☐ Foam ☐ Padding ☐ Foil ☐ Paper ☐ Unknown |
| (I) Surface area (mm^2^) [if applicable] |  |
| **MODEL-SPECIFIC PARAMETERS: Blast** | |
| (I) Device (vendor, make, model) |  |
| (H) Shock/blast tube: Diameter (cm:mm) |  |
| (H) Shock/blast tube: Length (cm:mm) |  |
| (H) Shock/blast tube: Exit end | ☐ Open ☐ Closed ☐ Unknown |
| (H) Position/orientation of animal | ☐ Prone ☐ Other ☐ Unknown |
| If other, please specify |  |
| (H) Animal restraint | ☐ Head ☐ Body ☐ Head and body ☐ Not restrained ☐ Unknown |
| (H) Shielding | ☐ Yes ☐ No ☐ Unknown |
| (I) If yes, please specify material | ☐ Metal ☐ Other ☐ Unknown |
| If other material, please specify |  |
| (H) If yes, please specify body part(s) |  |
| (H) Distance from blast (m:cm:mm) [if applicable] |  |
| (H) Pressure of exposure (kPa) [if applicable] |  |
| (H) Pressure of exposure (psi) [if applicable] |  |
| (I) Duration of exposure (sec:ms) [if applicable] |  |
| (H) Number of exposures [if applicable] |  |
| (H) Inter-exposure interval (days:min:hr) [if applicable] |  |
| **(H) MODEL-SPECIFIC PARAMETERS: CHI**  *Refer to* *Model-specific parameters: CCI* and/or *WD*. |  |
| **(H) MODEL-SPECIFIC PARAMETERS: Other**  *Upload relevant model-specific parameters and published reference, if available.* |  |
| **Model-specific information: Hypoxia and/or Ischemia** | |
| (H) Model specific information | ☐ Ischemic stroke model  ☐ HI injury model  ☐ Hypoxia-induced seizure model  ☐ Other |
| If other, please specify |  |
| (H) Induction method | ☐ Graded global hypoxia ☐ Unilateral ligation of common carotid artery ☐ Both ☐ Photothrombotic lesion ☐ Middle cerebral artery occlusion ☐ Other |
| If other, please specify |  |
| (H) If artery ligation, please specify side | ☐ Left ☐ Right ☐ Both ☐ Unknown |
| (Η) Ligation method | ☐ Suture ☐ Other ☐ Unknown |
| (Η) If suture, please specify type |  |
| (Η) If other ligation method, please specify |  |
| If hypoxia, please specify:  (H) O_2_ concentration (%; range) |  |
| (Η) Rate of O_2_ reduction (% per min) |  |
| (H) Duration of hypoxia (min) |  |
| (H) Nitrogen used | ☐ Yes ☐ No ☐ Unknown |
| If yes, please specify duration of infusion (min) |  |
| Device used (H) | ☐ Airtight chamber ☐ Oxygen meter ☐ Other ☐ Unknown |
| If other, please specify |  |
| (H) If hypoxia PLUS ischemia, please specify order | ☐ Ischemia first ☐ Hypoxia first  ☐ Unknown |
| (H) Please specify interval between insults (days:hr:min) |  |
| If photothrombotic lesion, please specify:  (H) Location (brain region, or coordinates) |  |
| (H) Dye used: Source (drug name + vendor) |  |
| (H) Dye used: Volume (ml.ul) |  |
| (H) Dye used: Concentration (mg/ml) |  |
| (H) Route of dye administration | ☐ i.v. right jugular ☐ i.v. left jugular ☐ Other ☐ Unknown |
| If other route of dye administration, please specify |  |
| (H) Light stimulation: diameter (mm) |  |
| (H) Light stimulation: intensity (mW) |  |
| (H) Light stimulation: duration (min:ss:ms) |  |
| (H) Type of light stimulation | ☐ Single stimulation ☐ Repeated  ☐ Unknown |
| (H) If repeated, please specify cycle number and frequency |  |
| (H) Stabilization method | ☐ Stereotaxic frame ☐ Hand-held  ☐ Restrained ☐ Other ☐ Unknown |
| If other, please specify |  |
| **Model-specific information: Hyperthermia models** | |
| (H) Hyperthermia induction methods | ☐ Heating lamp ☐ Mercury vapor lamp  ☐ Hot water bath ☐ Chamber of hot moist air ☐ Chamber of dry air ☐ Other |
| (I) If mercury vapor lamp, please specify (W) |  |
| (H) If other hyperthermia method, please specify |  |
| For each of the hyperthermia techniques, please define:  (H) Temperature (°C ; range) |  |
| (H) Duration (min) |  |
| (H) Kinetics (°C/min ramp) |  |
| (H) For temperature kinetics, please specify | ☐ Continuous temperature increase  ☐ Stepwise temperature increase ☐ Other ☐ Unknown |
| If other, please specify |  |
| (H) How are thermal measurements obtained?  *Refer to Physiological studies CRF modules for temperature monitoring (link form).* | ☐ Invasive ☐ Non-invasive ☐ Unknown |
| (H) If invasive, please specify | ☐ Ear probe ☐ Rectal probe ☐ Thermoregulatory probes ☐ Other  ☐ Unknown |
| If other, please specify |  |
| (H) If non-invasive, please specify | ☐ Biotelemetric devices ☐ Thermographic camera ☐ Other ☐ Unknown |
| If other, please specify |  |
| Additional chemical insult/challenge?  (e.g. lipopolysaccharide, LPS) | ☐ Yes ☐ No |
| *If yes, refer to ‘Model-specific information: infection-associated models’ in the Chemical models CRF for relevant data collection* |  |
| **Model specific information: Cortical freeze injury** | |
| (I) Probe: Source/ Vendor [if applicable] |  |
| (I) Probe: Material | ☐ Metal ☐ Other ☐ Unknown |
| If other material, please specify |  |
| (I) Probe: Shape | ☐ Circular ☐ Square ☐ Rectangular  ☐ Other ☐ Unknown |
| If other shape, please specify |  |
| (H) Probe: Diameter (mm) [if applicable] |  |
| (H) Temperature (° C; range) |  |
| (H) Duration (min:ss:ms) |  |
| (H) Depth | ☐ Surface contact ☐ Into tissue  ☐ Unknown |
| (H) If into tissue, please specify depth (mm) |  |
| (H) Nature of insult: Brain region | ☐ Frontal ☐ Parietal ☐ Other ☐ Unknown |
| (H) Nature of insult: Coordinates [if applicable] |  |
| (H) Nature of insult: Method | ☐ Manual/hand-held ☐ Stereotaxic controlled ☐ Unknown |
| (H) Nature of insult: Laterality | ☐ Left ☐ Right ☐ Bilateral ☐ Unknown |
| **Response to induction model / procedure** | |
| (H) Duration of apnea (sec) [if applicable] |  |
| (H) Latency to toe pinch response (sec) [if applicable] |  |
| (H) Latency to righting reflex (sec) [if applicable] |  |
| (H) Post-procedure fluid replenishment | ☐ Not administered ☐ Isotonic saline  ☐ Other ☐ Unknown |
| If other, please specify |  |
| (H) Volume (mL) |  |
| (H) Route of administration | ☐ i.p. ☐ s.c. ☐ i.v. ☐ Oral ☐ Other  ☐ Unknown |
| If other, please specify |  |
| (I) Post-procedure warmth provided | ☐ Yes ☐ No ☐ Unknown |
| If yes, please specify | ☐ Heat lamp ☐ Heat pad ☐ Other  ☐ Unknown |
| If other, please specify |  |
| (H) Post-impact dura impact | ☐ Yes ☐ No ☐ Unknown |
| (H) Acute seizures observed  *Refer to Seizure Phenotyping CRF (link form)* | ☐ Yes ☐ No ☐ Unknown |
| If yes, please provide details:  (H) When (hh:mm:ss) |  |
| (H) Duration (hh:mm:ss) |  |
| (H) Number of seizures observed |  |
| (H) Phenotype/classification |  |
| (H) Detection method | ☐ Behavioral ☐ EEG ☐ Both  ☐ Unknown |
| (H) Post-ictal observations | ☐ Normal behavior ☐ Inactive ☐ Hyperactive ☐ Other ☐ Unknown |
| If other post-ictal observations, please specify |  |
| (H) Acute procedure-related mortality of animal | ☐ Yes ☐ No ☐ Unknown |
| If yes, please specify:  (H) Date (MM/DD/YYYY) |  |
| (I) Time (hh:mm) |  |

**Abbreviations:** CRF: case report form; ID: identification; CDE: common data element; P: postnatal; MM: month; DD: day; YYYY: year; g: gram; mg: milligram; kg: kilogram; hh: hours; mm: minutes; ss: seconds; mL: millilitre; i.p.: intraperitoneal; i.v.: intravenous; s.c.: subcutaneous; i.n.: intranasal; i.m.: intramuscular; p.o.: per os; BNP: Bacitracin zinc, Neomycin sulfate, Polymycin B sulfate; SE: Status Epilepticus; TBI: traumatic brain injury; HI: hypoxic-ischemic; PMID: PubMed Identifier Number; PP: perforant path; Hz: hertz; hr: hour; min: minute; sec: second; CCI: controlled cortical impact; FPI: fluid percussion injury; WD: weight drop; CHI: closed head injury; mm: millimetre; #: number; °: degrees; ms: millisecond; m: metre; atm: atmosphere; cm: centimetre; kPa: kilopascal; psi: pound per square inch; O_2_: oxygen; %: percent; ul: microlitre; mW: milliwatt; W: watt; °C: degree Celsius; EEG: electroencephalography. Key for importance: H = high, I = intermediate and L = low priority.
