# Supplementary material for: A companion to the preclinical common data elements for rodent models of pediatric acquired epilepsy: A report of the TASK3‐WG1B, Pediatric and Genetic Models Working Group of the ILAE/AES Joint Translational Task Force
Source: Epilepsia Open. 2022 Oct 5;10(Suppl 1):S53–86. doi: 10.1002/epi4.12641 (PMC12375983; doi:10.1002/epi4.12641)
Supplement: Supplementary file 1 — Appendix S1 [file EPI4-10-S53-s001.zip › EPI4_12641_3. CRF module Chemical induction models.docx]

**Table 3:**

**Case Report Form:**

**Specific CRF 2 – Chemical induction models of early onset epilepsies and seizures in rodents**

Date that this CRF was filled out:

Name of person filling out CRF:

Project name/Identifier:

Animal ID:

Note: This form is designed to be completed per experimental procedure in a subset of experimental animals. Refer to **CORE CRF – Rodent Models of Pediatric Acquired Epilepsy** for cohort-based information (link form)

| **CDE Name** | **Data Collected** |
| --- | --- |

| Individual animal information | |
| --- | --- |
| (H) Animal identification method (ear tag, mark, etc.) | ☐ Yes ☐ No ☐ Unknown |
| If yes, please specify |  |
| (H) General health status, prior to model induction  *Refer to* *General Health Status CRF*  (link form) |  |

| **Induction model information** | |
| --- | --- |
| (H) Age at time of surgery/procedure/model induction [if applicable] *Note: P0 = day of birth.* |  |
| (H) Date of procedure (MM/DD/YYYY) |  |
| (H) Body weight (g) at time of procedure [if applicable] |  |
| (H) Anesthesia | ☐ Yes ☐ No ☐ Unknown |
| (H) If anesthesia was administered, please specify type | ☐ Isoflurane ☐ Ketamine/xylazine ☐ Other ☐ Unknown |
| If other type of anesthesia, please specify |  |
| (H) Anesthesia induction dose | ☐ % ☐ mg/kg ☐ Unknown |
| (H) Maintenance dose | ☐ % ☐ mg/kg ☐ Unknown |
| (H) Route of anesthesia administration | ☐ Inhaled ☐ Injected ☐ Other ☐ Unknown |
| If other route of anesthesia administration, please specify |  |
| Duration of anesthesia (hh:mm:ss) |  |
| (H) Analgesic drugs (H) | ☐ Yes ☐ No ☐ Unknown |
| If analgesics were administered, please specify:  (H) Name of drug *Check all applicable.* | ☐ Acetaminophen ☐ Buprenorphine ☐ Ibuprofen ☐ Meloxicam ☐ Other  ☐ Unknown |
| If other analgesics used, please specify |  |
| For each of the analgesics used, please specify:  (H) Date analgesic drug given (MM/DD/YYYY) |  |
| (I) Time analgesic drug given (hh:mm:ss) |  |
| (I) Injection volume (mL) |  |
| (H) Dose (mg/kg) |  |
| (H) Route of administration | ☐ Intraperitoneal (i.p.) ☐ Intravenous (i.v.) ☐ Subcutaneous (s.c.) ☐ Intranasal (i.n.) ☐ Intramuscular (i.m.) ☐ Oral (p.o.) ☐ Topical ☐ Implanted mini-pump ☐ Other  ☐ Unknown |
| If other route of administration, please specify |  |
| (H) Antibiotic drugs | ☐ Yes ☐ No ☐ Unknown |
| If antibiotics were administered, please specify:  (H) Name of drug *Check all applicable.* | ☐ Amoxicillin ☐ BNP ☐ Doxycycline  ☐ Tetracycline ☐ Other ☐ Unknown |
| If other antibiotics used, please specify |  |
| For each of the antibiotics used, please specify:  (H) Date antibiotic drug given (MM/DD/YYYY) |  |
| (I) Time antibiotic drug given (hh:mm:ss) |  |
| (I) Injection volume (mL) |  |
| (H) Dose (mg/kg) |  |
| (H) Route of administration | ☐ Intraperitoneal (i.p.) ☐ Intravenous (i.v.) ☐ Subcutaneous (s.c.) ☐ Intranasal (i.n.) ☐ Intramuscular (i.m.) ☐ Oral (p.o.) ☐ Topical ☐ Implanted mini-pump ☐ Other  ☐ Unknown |
| If other route of administration, please specify |  |
| (H) Type of model | ☐ Models of epileptic spasms  ☐ Models of seizures  ☐ Models of status epilepticus (SE)  ☐ Infection-associated models  ☐ Other |
| If other, please specify |  |
| (I) Published reference for model | ☐ Yes ☐ No ☐ Unknown ☐ Not published |
| (H) If published reference model known, please specify (PMID) |  |
| (H) Monitoring during procedure  *Refer to Physiological studies CRF modules for temperature, respiration, heart rate, and blood pressure monitoring (link form).* |  |
| **Model-specific information: epileptic spasms** | |
| (H) Specific model of epileptic spasms  *Check all applicable*. | ☐ N-methyl-D-aspartate (NMDA) model  ☐ Gamma-Butyrolactone (GBL) model of epileptic spasms in Down Syndrome  ☐ Predisposing condition / postnatal NMDA  ☐ Multiple-hit rat model of Infantile Spasms (IS)  ☐ Tetrodotoxin (TTX) model of epileptic spasms and hypsarrhythmia  ☐ Genetic etiology model  ☐ Other |
| If other or genetic, please specify  *For genetic models please link to the CRFs on genetic etiology models of seizures.* |  |
| (H) Predisposing conditions  *Check all applicable.* | ☐ Pharmacological/chemical  ☐ Perinatal Stress  ☐ Surgical procedures (e.g., adrenalectomy)  ☐ Malformations  ☐ Other  ☐ None |
| If other, please specify |  |
| For each one of the above choices please specify:  (H) Date/age range |  |
| (H) Agent or mechanism used |  |
| (H) Choice of drug/agent used for induction  *Check all applicable.* | ☐ NMDA  ☐ Gamma-butyrolactone (GBL)  ☐ Doxorubicin (DOX)  ☐ Lipopolysaccharide (LPS)  ☐ P-chlorophenylalanine (PCPA)  ☐ Tetrodotoxin (TTX)  ☐ Methylazoxymethanol  ☐ Other |
| If other, please specify |  |
| (H) Drug administration protocol | ☐ Single injections ☐ Repeated injections ☐ Other ☐ Unknown |
| If other, please specify |  |
| For each of the drugs/agents used, please specify:  (Note: Repeat sections for each drug/agent used. For different doses refer to *CRF module 1: General Core Pharmacology Dosing records.*)  (H) Date given (MM/DD/YYYY) |  |
| (I) Time given (hh:mm:ss) |  |
| (I) Injection volume (mL) |  |
| (H) Dose (mg/kg) |  |
| (H) Vehicle used | ☐ Yes ☐ No ☐ Unknown |
| (H) Route of administration | ☐ i.p. ☐ i.v. ☐ s.c. ☐ i.n. ☐ i.m. ☐ Oral (p.o.) ☐ i.c.v. ☐ Implanted mini-pump  ☐ Other ☐ Unknown |
| If other route of administration, please specify |  |
| (H) If intracranial administration, which side was injected? | ☐ Right hemisphere ☐ Left hemisphere  ☐ Bilateral ☐ Unknown |
| (H) Please specify brain region  Stereotaxic coordinates:  *Use (-) to indicate posterior* | Anterior to Bregma_____  Anterior to Lambda ________  Right_________  Left_________  Depth______ |
| If mini-pump, please specify:  (I) Model/ Source |  |
| (H) Date minipump placed (MM/DD/YYYY) |  |
| (I) Time minipump placed (hh:mm:ss)  *If time unknown, use 00:00:00* |  |
| (H) Date minipump removed (MM/DD/YYYY) |  |
| (I) Time minipump removed (hh:mm:ss)  *If time unknown, use 00:00:00* |  |
| (H) Minipump location |  |
| (H) Minipump drug dose (mg/kg) |  |
| (H) Infusion location of the implanted pump | ☐ i.p. ☐ s.c. ☐ i.c.v. ☐ Other ☐ Unknown |
| If other, please specify |  |
| **Model-specific information: induced seizures and SE** | |
| (H) Specific model of seizures and SE | ☐ Kainate model of SE  ☐ Pilocarpine-induced SE  ☐ NMDA-induced SE  ☐ Pentylenetetrazol (PTZ) model  ☐ Flurothyl-induced seizures  ☐ Tetanus toxin-induced seizures  ☐ Corticotropin-releasing hormone (CRH)-induced seizure model  ☐ Bicuculline-induced model  ☐ Other |
| If other, please specify |  |
| (H) Choice of drug/agent used for induction | ☐ Kainic acid  ☐ Pilocarpine  ☐ NMDA  ☐ Pentylenetetrazol (PTZ)  ☐ Flurothyl  ☐ Tetanus toxin  ☐ Corticotropin-releasing hormone (CRH)-  ☐ Bicuculline  ☐ Other |
| If other, please specify |  |
| (H) Pretreatment drug/agent used | ☐ Lithium  ☐ Lithium chloride  ☐ Homocysteine  ☐ Methylscopolamine  ☐ Atropine methyl bromide  ☐ Other  ☐ None |
| If other, please specify |  |
| (H) Drug administration protocol | ☐ Single injections ☐ Repeated injections ☐ Other ☐ Unknown |
| If other, please specify |  |
| For each of the drugs/agents used, please specify:  (Note: Repeat sections for each drug/agent used. For different doses refer to *CRF module 1: General Core Pharmacology Dosing records.*)  (H) Date given (MM/DD/YYYY) |  |
| (I) Time given (hh:mm:ss) |  |
| (I) Injection volume (mL) |  |
| (H) Dose (mg/kg) |  |
| (H) Vehicle used | ☐ Yes ☐ No ☐ Unknown |
| (H) Route of administration | ☐ i.p. ☐ i.v. ☐ s.c. ☐ i.n. ☐ i.m. ☐ Oral (p.o.) ☐ i.c.v. ☐ Implanted mini-pump ☐ Other ☐ Unknown |
| If other route of administration, please specify |  |
| (H) If intracranial administration, which side was injected? | ☐ Right hemisphere ☐ Left hemisphere ☐ Bilateral ☐ Unknown |
| (H) Please specify brain region and stereotaxic coordinates |  |
| If mini-pump, please specify:  (I) Model/ Source |  |
| (H) Date minipump placed (MM/DD/YYYY) |  |
| (H) Time minipump placed (hh:mm:ss)  *If time unknown, use 00:00:00* |  |
| (H) Date minipump removed (MM/DD/YYYY) |  |
| (H) Time minipump removed (hh:mm:ss)  *If time unknown, use 00:00:00* |  |
| (H) Minipump location |  |
| (H) Minipump drug dose (mg/kg) |  |
| (H) Infusion location of the implanted pump | ☐ i.p. ☐ s.c. ☐ i.c.v. ☐ Other ☐ Unknown |
| If other, please specify |  |
| **Model-specific information: infection-associated models** | |
| (H) Specific infection-associated model | ☐ Maternal immune activation (MIA) model  ☐ Herpes Simplex Virus (HSV)-induced model  ☐ Neurocysticercosis model of limbic seizures  ☐ Other |
| If other, please specify |  |
| (H) Choice of drug/agent/pathogen used for induction  *Check all applicable.* | ☐ LPS  ☐ Poly (I:C)  ☐ Substrain of HSV-1  ☐ *T. solium* oncospheres  ☐ *T. crassiceps*  ☐ Other |
| If other, please specify |  |
| (H) Drug administration protocol | ☐ Single injections ☐ Repeated injections ☐ Other ☐ Unknown |
| If other, please specify |  |
| For each of the drugs/agents/pathogens used, specify:  (Note: Repeat sections for each drug/agent/pathogen used. For different doses refer to *CRF module 1: General Core Pharmacology Dosing records.*)  (H) Date given (MM/DD/YYYY) |  |
| () Time given (hh:mm:ss) |  |
| (I) Injection volume (mL) |  |
| (H) Dose (mg/kg) |  |
| (H) Infection dose (TCID_50_, PFU, number of oncospheres, etc) |  |
| (H) Vehicle used | ☐ Yes ☐ No ☐ Unknown |
| (H) Route of administration | ☐ i.p. ☐ i.v. ☐ s.c. ☐ i.n. ☐ i.m. ☐ Oral (p.o.) ☐ i.c.v. ☐ Implanted mini-pump  ☐ Other ☐ Unknown |
| If other route of administration, please specify |  |
| (H) If intracranial administration, which side was injected? | ☐ Right hemisphere ☐ Left hemisphere  ☐ Bilateral ☐ Unknown |
| (H) Please specify brain region and stereotaxic coordinates |  |
| If mini-pump, please specify:  (I) Model/ Source |  |
| (H) Date minipump placed (MM/DD/YYYY) |  |
| (H) Time minipump placed (hh:mm:ss)  *If time unknown, use 00:00:00* |  |
| (H) Date minipump removed (MM/DD/YYYY) |  |
| (H) Time minipump removed (hh:mm:ss)  *If time unknown, use 00:00:00* |  |
| (H) Minipump location |  |
| (H) Minipump drug dose (mg/kg) |  |
| (H) Infusion location of the implanted pump | ☐ i.p. ☐ s.c. ☐ i.c.v. ☐ Other ☐ Unknown |
| If other, please specify |  |
| **Response to induction model/procedure** | |
| (H) Post-procedure fluid replenishment | ☐ Not administered ☐ Isotonic saline  ☐ Other ☐ Unknown |
| If other, please specify |  |
| (H) Volume (mL) |  |
| (H) Route of administration | ☐ i.p. ☐ s.c. ☐ i.v. ☐ Oral ☐ Other  ☐ Unknown |
| If other, please specify |  |
| (I) Post-procedure warmth provided | ☐ Yes ☐ No ☐ Unknown |
| If yes, please specify | ☐ Heat lamp ☐ Heat pad ☐ Other  ☐ Unknown |
| If other, please specify |  |
| (H) Acute post-impact seizures observed  *Refer to Seizure Phenotyping CRF (link form)* | ☐ Yes ☐ No ☐ Unknown |
| If yes, please provide details:  (H) Time (hh:mm:ss) |  |
| (H) Duration (hh:mm:ss) |  |
| (H) Number of seizures observed |  |
| (H) Phenotype/classification |  |
| (H) Detection method | ☐ Behavioral ☐ EEG ☐ Both ☐ Unknown |
| (H) Post-ictal observations | ☐ Normal behavior ☐ Inactive ☐ Hyperactive ☐ Other ☐ Unknown |
| If other post-ictal observations, please specify |  |
| (H) Acute procedure-related mortality of animal | ☐ Yes ☐ No ☐ Unknown |
| If yes, please specify:  (H) Date (MM/DD/YYYY) |  |
| (I) Time (hh:mm:ss) |  |

**Abbreviations:** CRF: case report form; ID: identification; CDE: common data element; P: postnatal; MM: month; DD: day; YYYY: year; g: gram; mg: milligram; kg: kilogram; hh: hours; mm: minutes; ss: seconds; mL: millilitre; i.p.: intraperitoneal; i.v.: intravenous; s.c.: subcutaneous; i.n.: intranasal; i.m.: intramuscular; p.o.: per os; SE: Status Epilepticus; PMID: PubMed Identifier Number; NMDA: N-methyl-D-aspartate; GBL: Gamma-Butyrolactone; IS: infantile spasms; TTX: tetrodotoxin; DOX: doxorubicin; LPS: lipopolysaccharide; PCPA: p-chlorophenylalanine; i.c.v.: intracerebroventricular; PTZ: pentylenetetrazol; CRH: corticotropin-releasing hormone; MIA: maternal immune activation; HSV: herpes simplex virus; Poly (I:C): polyinosinic-polycytidylic acid; T.: taenia; TCID_50_: 50% tissue culture infectious dose; PFU: plaque forming units. Key for importance: H = high, I = intermediate and L = low priority.
